# Research on the Optimization of Uncertain Multi-Stage Production Integrated Decisions Based on an Improved Grey Wolf Optimizer

**DOI:** 10.3390/biomimetics10110775

**Published:** 2025-11-15

**Authors:** Weifei Gan, Xin Zhou, Wangyu Wu, Chang-An Xu

**Affiliations:** 1 School of Computer and Information Engineering, Institute for Artificial Intelligence, Shanghai Polytechnic University, Shanghai 201209, China; 2 School of Computer and Information Engineering, Shanghai Polytechnic University, Shanghai 201209, China; 20221113029@stu.sspu.edu.cn; 3 School of Computer Science, University of Liverpool, Liverpool L69 3DR, UK; 4 College of Materials and Energy, South China Agricultural University, Guangzhou 510642, China; xuchangan@scau.edu.cn

**Keywords:** bio-inspired intelligence, defect-rate uncertainty, improved grey wolf optimizer, multi-stage production optimization

## Abstract

Defect-rate uncertainty creates cascading operational challenges in multi-stage production, often driving inefficiency and misallocation of labor, materials, and capacity. To confront this, we develop a multi-stage Production Integrated Decision (MsPID) framework that unifies quality inspection and shop-floor decision-making within a single computational model. The framework couples a two-stage sampling inspection policy—used to statistically learn and control defect-rate uncertainty via estimation and rejection rules—with a multi-process, multi-part production decision model. Optimization is carried out with an Improved Grey Wolf Optimizer (IGWO) enhanced with Latin hypercube sampling (LHS) for uniformly diverse initialization; an evolutionary factor mechanism that blends simulated binary crossover (SBX) among three leadership-guided parents (Alpha, Beta, Delta) to strengthen global exploration in early iterations and focus exploitation later; and a greedy, mutation-assisted opposition learning step applied to the lowest-performing quartile of the population to effect leader-informed local refinement and accept only fitness-improving moves. Experiments show the method identifies minimum-cost policies across six single-stage benchmark cases and yields a total profit of 43,800 units in a representative multi-stage scenario, demonstrating strong performance in uncertain environments. Sensitivity analysis further clarifies how recommended decisions adapt to shifts in estimated defect rates, finished product prices, and swap/changeover losses. These results highlight how bio-inspired intelligence can enable adaptive, efficient, and resilient integrated production management at scale.

## 1. Introduction

Since its inception, the Industrial Revolution has epitomized an unprecedented confluence of science and technology within the industrial domain, radically reshaping the modes of human existence, labor, and social engagement [1]. With the dawn of the Fourth Industrial Revolution, conventional industries are confronting disruptive transformations driven by a wave of technological innovations. In reaction, a growing number of enterprises have turned to artificial intelligence (AI) to develop adaptive industrial systems. Through systematic intelligent enhancements, AI furnishes more sophisticated and efficient solutions to challenges across supply chains, manufacturing processes, and decision-making frameworks [2,3].

Nonetheless, traditional industrial production continues to be plagued by persistent concerns regarding process quality and exorbitant inspection costs, compelling firms to pursue heightened operational efficiency, cost reduction, and unwavering quality assurance. Missbauer H. has emphasized the transformative potential of information and computational technologies in unlocking novel applications within production environments [4]. Similarly, Tseng M.L. has drawn attention to the inefficiencies and environmental drawbacks of obsolete automation systems, underscoring the imperative for modernization [5]. Expanding on these observations, Khan S.A.R. has advocated for the optimization of production workflows as a viable strategy to mitigate these issues [6].

Consequently, the reconciliation of process reliability with economic efficiency has emerged as a paramount research imperative in industrial studies [7]. While extant literature frequently emphasizes the optimization of discrete manufacturing stages, Alvandi et al. contend that an integrated modeling approach—one that holistically unifies optimization throughout the entire decision-making continuum—can concurrently elevate manufacturing performance and curtail operational expenditures [8].

This study establishes a Multi-stage Production Integrated Optimization (MsPIO) model to tackle complex production planning and control challenges under uncertainty. By leveraging artificial intelligence and systems engineering principles, the proposed approach facilitates the development of integrated solutions that enhance resource efficiency through synchronized cross-process coordination. The principal contributions of this work are threefold:

1. Development of an Integrated Multi-stage Production Optimization Model: The MsPIO model systematically unifies a two-stage sampling inspection mechanism—designed for defect-rate estimation and criterion-based decision-making—with multi-process, multi-component production planning. This integrated framework provides a coherent structure for addressing uncertainty and variability in complex production systems, aligning with systemic approaches to operational decision-making.
2. Design of an Enhanced Metaheuristic Solution Strategy: An Improved Grey Wolf Optimizer (IGWO) is introduced, incorporating Latin hypercube sampling to ensure uniform initialization of the population. The algorithm further integrates an evolutionary factor mechanism based on simulated binary crossover (SBX) and three leadership-guided parents (Alpha, Beta, Delta) to strengthen global exploration. A greedy mutation-based opposition learning strategy is applied to the lowest-performing quarter of the population, enabling effective local refinement and accelerating convergence toward high-quality solutions.
3. Comprehensive Experimental Validation and Sensitivity Analysis: Extensive experiments validate the model’s effectiveness and robustness under both cost-minimization and profit-maximization objectives. Results demonstrate that the proposed IGWO-based method not only identifies cost-optimal strategies across multiple single-stage production configurations but also achieves a total profit of 43,800 in a multi-stage production scenario. Through systematic sensitivity analysis, the study elucidates how key parameters—such as estimated defect rates (modulated by confidence levels), finished product price fluctuations, and replacement losses—influence optimal decisions. These insights offer valuable guidance for intelligent production management in environments shaped by mass customization and operational uncertainty.

The remainder of this paper is structured as follows. Section 2 reviews related work and the background of the cited models. Section 3 describes our designed sampling inspection mechanism and the multi-stage decision-making model for multiple processes and multiple parts based on the Improved Grey Wolf Optimizer (IGWO), which leverages defect-rate estimation to optimize multi-stage production decisions. Section 4 presents the experimental results, including algorithmic convergence analysis, production optimization performance, comparisons of optimal decision schemes, and sensitivity analysis. Section 5 concludes the paper.

## 2. Related Work

With the advent of Industry 4.0, the manufacturing sector is steadily transforming into smart production, leveraging AI, big-data analytics, and cloud computing to manage and optimize every stage of the production lifecycle—thereby boosting production efficiency and precision while reducing operating costs [9,10]. Recent studies show that large, well-capitalized firms deploying these advanced technologies consistently outperform small- and medium-sized enterprises and achieve marked improvements in production-flow efficiency [11,12]. Moreover, smart-optimization techniques have proven both effective and widely applicable across diverse industries [13,14,15], underscoring the necessity of optimizing production processes.

When defect-rate uncertainty arises, it must first be quantified via estimation [16]. Most scholars model the basic conditions with a binomial distribution and approximate its probabilities by simple random sampling—an approach prized for its ease of implementation [17]—though recent work has produced refined binomial sampling variants [18,19]. To better capture real-world complexity and avoid sampling bias, hypergeometric sampling (i.e., sampling without replacement) can augment the binomial approach and yield estimates closer to the true defect rate [20,21]. Some researchers have even applied this method to estimate sharding-failure probabilities in blockchain systems [22]; however, this method can only estimate the probability, whereas uncertainty calls for a confidence-interval framework. The Clopper–Pearson method provides exactly such an efficient, reasonable and lightweight interval estimate, defining an uncertainty band around the defect-rate estimate according to a prescribed confidence level—without imposing undue time or computational burdens [23,24]. Together, these techniques transform an uncertain defect rate into a confidence interval that can be fed into an optimization model for robust decision-making.

Beyond front-end inspection methods, the downstream “decision” phase determines an enterprise’s ultimate production cost. Schärer emphasizes that optimizing production decision-making in enterprises must account for process-related costs and requires more robust algorithms to reduce expenditures [25]. This calls for optimization approaches that consider global solutions, a task for which metaheuristic algorithms are particularly well-suited [26]. Recent advancements have further expanded the toolbox for tackling such challenges. For instance, the Schrödinger optimizer introduces a novel quantum duality-driven mechanism, demonstrating strong performance in stochastic optimization and complex engineering problems [27]. Similarly, significant improvements in Particle Swarm Optimization (PSO) have been achieved through the integration of quadratic interpolation and a new local search approach, enhancing its precision in parameter estimation tasks [28]. Enhancements to other popular algorithms, such as the Moth Flame Optimizer incorporating local escape operators [29] and gradient-based methods refined with quasi-Newton rules and new local search techniques [30], also exemplify the ongoing trend of hybridizing and refining metaheuristics to balance global exploration with local exploitation. Silva, Tao, and colleagues have applied such metaheuristic algorithms to production decision-making to address cost-related challenges. Their findings indicate that these methods enable effective evaluation and optimization of overall costs, ensuring reductions across various production stages and leading to minimized total cost [31,32]. In contrast, conventional optimization techniques often suffer from premature convergence to local optima. Therefore, appropriate algorithmic enhancements are necessary to improve solution quality. For instance, Liu et al. improved the Crested Porcupine Optimization algorithm, achieving notable results in optimizing delivery routes for unmanned aerial vehicles by reducing angular variations and path length, thereby enhancing distribution efficiency and lowering operational costs [33]. Similarly, Zhang et al. refined the Ivy algorithm by integrating principles from particle swarm optimization, which improved its performance in handling numerous hyperparameters in neural networks. This enhancement allowed for balanced global exploration and precise parameter tuning, ultimately increasing predictive accuracy [34]. Additionally, Qiu and colleagues advanced the grey wolf optimization algorithm to address complex numerical global optimization problems in engineering design, providing new impetus for production optimization [35]. Extending GWO to other infrastructure settings, Sujono and Musafa hybridized Grey Wolf Optimization with the Whale Optimization Algorithm for load-shedding in isolated distribution networks, illustrating how cross-metaheuristic design can improve decision robustness under stringent operational constraints [36]. Likewise, Fauzan, Munadi, Sumaryo, and Nuha proposed an enhanced GWO for transmission-power optimization in wireless sensor networks, underscoring that careful operator redesign and parameter control can yield more energy-efficient configurations without sacrificing network performance [37]. Adopting a similar improvement-oriented approach, Musshoff et al. optimized production processes by identifying optimal decisions that significantly cut costs, thereby supporting sustainable enterprise operations through enhanced operational efficiency [38]. After that, Pan et al. developed a four-step decision optimization model and improved the grey wolf optimization algorithm, called the Decision Grey Wolf Optimization Algorithm (DGWO) [39]. This method solves the global optimal decision, significantly improves the global optimization performance of the decision, and ensures that the performance indicators of multiple decision problems rank at the top, which means that this method has been fully applied to decision optimization.

**Figure 1:**
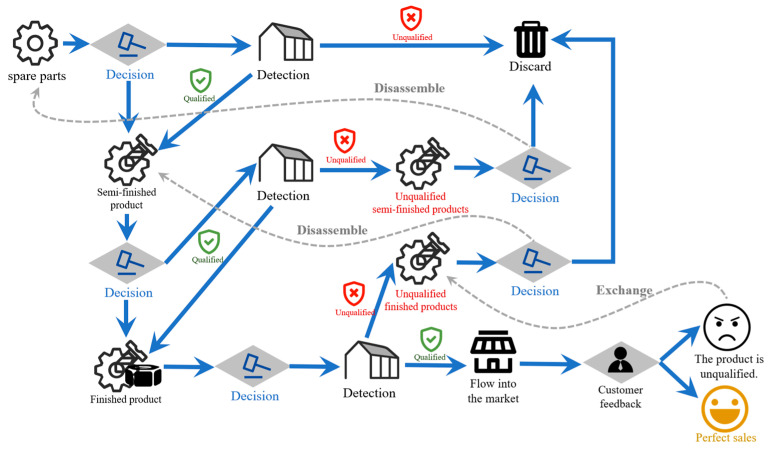
Complete model flow chart with multiple processes and multiple parts.

To resolve our research problems, we propose a Multi-stage Production Integrated Optimization (MsPIO) model grounded in statistical process control. First, a defect-rate prediction model is built using hypergeometric distribution point estimation and Clopper–Pearson exact confidence intervals, enabling dynamic assessment of process quality equilibrium through sampling data. Building on this, a coordinated decision-making framework for multiple processes and parts is constructed (visually summarized in Figure 1), where, Red text represents states or products that are unqualified/defective (e.g., “Unqualified,” “Unqualified semi-finished products,” “Unqualified finished products”). Green text represents states or products that are qualified/conforming and allowed to proceed to the next stage. Yellow text represents final ideal outcome, i.e., successful sales and satisfied customers (“Perfect sales”). Grey text represents auxiliary processes or annotations (e.g., “Disassemble,” “Exchange”) that describe supporting actions rather than main product states. Blue lines represent the main forward flow of products through the manufacturing, inspection and sales process. Grey (dashed) lines represent feedback and rework paths, such as disassembly, reuse of parts, or customer returns/exchanges, which are secondary to the main process flow. After that, IGWO establishes a synchronization mechanism for process parameters, achieving globally optimal decisions that maximize manufacturing-system symmetry. Finally, global sensitivity analysis reveals how key parameters—confidence thresholds, product pricing, and process-change costs—disrupt or preserve systemic balance through controlled perturbation experiments.

## 3. Methodologies

Before we start the methodology, we need to define our symbol names and descriptions, as shown in Table 1:

This study focuses on sampling inspection and robust decision optimization in the multi-stage production of electronic products and proposes an MsPIO. The model simulates the sampling process to estimate defect rates at each process stage and embeds these estimates into a Grey Wolf Optimizer–driven decision-making model for multiple processes and multiple parts, thereby enabling stochastic adjustment and optimization of assembly, inspection, and disassembly strategies.

### 3.1. Model Assumptions

The model assumptions are particularly important during the experimental process, reflecting the practical constraints of multi-stage optimization through the following assumptions:

1. It is assumed that the qualification of each sample is independent.
2. It is assumed that the enterprise’s quality inspection system is accurate and error-free.
3. It is assumed that all finished products entering the market will be successfully sold.

### 3.2. Two-Stage Sampling Inspection Model

Inspired by the “quick reject, strict accept” strategy, we establish a two-stage sampling model to more effectively control both Type I error α and Type II error β. In the early stage, obviously defective batches are rapidly rejected; in the later stage, compliant batches are strictly accepted. This approach minimizes the sample size while maintaining control over both error types. To address the uncertainty of defect rates in practice, we further incorporate binomial point estimates [32] and Clopper–Pearson confidence intervals [39] to perform confidence inference on the overall defect rate, thereby enhancing decision robustness and interpretability.

#### 3.2.1. Simulation of Product Sequence

Let the total number of items in a batch be *N*, of which *Q* are actually defective, yielding a true defect rate of p=Q/N. For quality control, a nominal defect rate $p_{0}$ is set, and a threshold $Q_{0}=\left\lfloor Np_{0}\right\rfloor$ of the number of defective products is introduced as the worst case. If $Q>Q_{0}$, the batch of products is considered to be defective.

In practice, full inspection of the entire batch is infeasible, so we perform sampling inspection. From the *N* items, we randomly draw *n* samples without replacement for inspection. Let *X* denote the number of defectives observed in the sample. Because sampling is without replacement and each item’s quality status is predetermined; therefore, *X* follows the hypergeometric distribution of *N*, *Q*, and *n*, denoted as:

(1) X ~ Hypergeometric(N,Q,n),

The probability mass function is:

(2) $$P(X=k)=\frac{\left(\begin{array}{c} Q\\ k \end{array}\right)\left(\begin{array}{c} N-Q\\ n-k \end{array}\right)}{\left(\begin{array}{c} N\\ n \end{array}\right)}\;,\;\max(0,n-N+Q)\leq k\leq \min(n,Q).$$

#### 3.2.2. Stage Two: Acceptance Test

The model design introduces a “quick rejection test” mechanism in the first stage. The primary goal of this stage is to make a swift decision to reject obviously nonconforming lots, thereby avoiding resource wastage in subsequent stages. Let the initial sample size be $n_{1}$ and the rejection threshold be *q*. The decision process is as follows:

1. If the number of defectives in the first-stage sample is $X_{1}>q$, the lot is immediately rejected.
2. If $X_{1}\leq q$, the lot moves to the second stage for further inspection.

To ensure that the rejection decision in the first stage is statistically robust, the probability of a Type I error must be strictly controlled. At this point, the control condition for Type I error is:

(3) $$P(X_{1}>q\;|\;p=p_{0})\leq \alpha .$$

#### 3.2.3. Stage One: Quick Rejection Test

If a lot is not rejected in the first stage, it moves to the second stage for a more stringent acceptance test. At this point, the $n_{1}$ samples already drawn remain unchanged, and $n_{2}$ additional samples are drawn from the remaining $N-n_{1}$ products, resulting in a total sample size of $n_{z}=n_{1}+n_{2}$. The observation result for the second stage is the number of defective products, $X_{2}$, and the final total number of defective products is $X_{z}=X_{1}+X_{2}$. The decision process is as follows:

1. If $X_{z}\leq c$, the lot is accepted.
2. If $X_{z}>c$, the lot is rejected.

To control the risk of Type II errors, the following condition must be satisfied:

(4) $$P(X_{z}\leq c\;|\;p=p_{1})\leq \beta ,$$

where $p_{1}$ is the unacceptable defective rate.

#### 3.2.4. Estimation and Confidence Prediction Under Uncertain Defective Rate

Due to the inherent randomness of the sampling process and the uncertainty of the defective rate itself, relying solely on point estimates can lead to misjudgment. Therefore, a more robust confidence inference method is introduced to assess the uncertainty range of the defective rate.

After sampling, we get $n_{z}$ and $X_{z}$, and the point estimate of the defective rate is naturally:

(5) $$\overset{⌢}{p}=\frac{X_{z}}{n_{z}},$$

Proposed by Cochran’s classic sampling method, the hypergeometric distribution is generally considered to perform well when the sample size *n* is less than 5% of the population size *N* [40]. In this thesis, *N* is 500, *n* is 22, and the sampling ratio is 4.4%. The study meets specific application conditions; therefore, the hypergeometric distribution can be well approximated by the binomial distribution in this research. Based on the binomial distribution assumption, the Clopper–Pearson method is used in this paper to construct a confidence interval for the true defective rate *p*, suitable for small sample scenarios. Let the confidence level be 1−γ, then the confidence interval [pL,pU] for the true defective rate *p* can be given by the following formulas:

- Lower confidence bound *pL*

(6) $$pL=BetaInv(\frac{\gamma }{2},X_{z},n_{z}-X_{z}+1),$$

- Upper confidence bound *pU*

(7) $$pU=BetaInv(1-\frac{\gamma }{2},X_{z}+1,n_{z}-X_{z}),$$

where BetaInv(a,b,c) represents the *a*-quantile of the *Beta* distribution, with parameters *b* and *c*.

### 3.3. Decision-Making Model for Multiple Processes and Multiple Parts

#### 3.3.1. Decision Variables

Let $D_{k}$ be a binary variable that indicates whether to inspect a spare part or to inspect and disassemble the finished product; its detailed meaning is given below.

#### 3.3.2. Objective Function

- Spare Parts Inspection Stage

In this stage, we must decide whether to inspect each spare part. If inspection is carried out, all nonconforming spare parts are discarded immediately; if inspection is skipped, uninspected spare parts—potentially including defective ones—proceed directly to assembly and may adversely affect final product quality.

Introduce a decision variable $D_{1}$, representing whether to inspect the spare parts, that is:

(8) $$D_{1}=\left\{\begin{array}{l} 0,\;\mathrm{when}\;\mathrm{the}\;\mathrm{components}\;\mathrm{are}\;\mathrm{not}\;\mathrm{inspected}\\ 1,\;\mathrm{when}\;\mathrm{the}\;\mathrm{components}\;\mathrm{are}\;\mathrm{inspected} \end{array}\right.,$$

When $D_{1}=0$ occurs, spare parts are not inspected and are directly assembled. In this case, the cost consists only of the purchasing cost, namely:

(9) $$C_{\mathrm{lg}}=Q_{i}u_{i},$$

where $C_{\mathrm{lg}}$ denotes the purchasing cost of the spare parts, $Q_{i}$ represents the quantity of the *i*-th type of spare part, and $u_{i}$ indicates the unit purchase price of the *i*-th spare part.

When $D_{1}=1$ occurs, additional inspection costs are incurred. Defective spare parts are discarded, and only qualified spare parts are assembled. In this case, the cost becomes:

(10) $$C_{\mathrm{lg}}+C_{lj}=Q_{i}(u_{i}+d_{i}),$$

where $C_{lj}$ represents the inspection cost of the spare parts, and $d_{i}$ denotes the inspection cost of the *i*-th type of spare part.

To summarize, the total cost at this stage includes both the purchasing cost and the inspection cost of the spare parts:

(11) $$C_{\mathrm{lg}}+C_{lj}=Q_{i}(u_{i}+D_{1}d_{i})\;.$$

- Finished Product Inspection Stage

In this stage, it is necessary to assess whether the finished products should undergo quality inspection. During the inspection process, defective products will enter the disassembly process, while qualified products and uninspected finished products, including potential defects, will directly enter the market for distribution.

Introduce a decision variable $D_{2}$, which represents whether to inspect the finished products, namely:

(12) $$D_{2}=\left\{\begin{array}{l} 0,\;\mathrm{when}\;\mathrm{the}\;\mathrm{finished}\;\mathrm{product}\;\mathrm{is}\;\mathrm{not}\;\mathrm{inspected}\\ 1,\;\mathrm{when}\;\mathrm{the}\;\mathrm{finished}\;\mathrm{product}\;\mathrm{is}\;\mathrm{inspected} \end{array}\right.,$$

When $D_{2}=0$ occurs, the finished product is assembled and directly enters the market without inspection. The cost consists of the assembly cost and the sales revenue, namely:

(13) $$C_{cz}+C_{cs}=Q_{c}(a_{c}-S_{c}),$$

where $C_{cz}$ represents the assembly cost of the finished product, $C_{cs}$ denotes the sales revenue of the finished product, $Q_{c}$ represents the quantity of finished products, $a_{c}$ represents the assembly cost per finished product, and $S_{c}$ represents the market price of a finished product.

When $D_{2}=1$ occurs, in addition to the assembly cost, defective finished products only require inspection cost before entering the disassembly stage, while qualified finished products enter the market for sale and generate revenue. In this case, the cost consists of the assembly cost, inspection cost, and sales revenue, namely:

(14) $$C_{cz}+C_{cj}+C_{cs}=Q_{c}a_{c}+Q_{c}d_{c}-Q_{c}(1-P_{c})S_{c},$$

where $C_{cj}$ represents the inspection cost of the finished product, $d_{c}$ denotes the inspection cost per finished product, and $P_{c}$ represents the defect rate of the finished products.

To summarize, the total cost at this stage consists of the assembly cost, inspection cost, and sales revenue of the finished product, namely:

(15) $$C_{cz}+C_{cj}+C_{cs}=Q_{c}\left[\left(a_{c}-S_{c})+D_{2}(d_{c}+P_{c}S_{c}\right)\right]\;.$$

- Defective Finished Product Disassembly Stage

Introduce a decision variable $D_{3}$, which represents whether to disassemble defective finished products, namely:

(16) $$D_{3}=\left\{\begin{array}{l} 0,\;\mathrm{when}\;\mathrm{the}\;\mathrm{defective}\;\mathrm{finished}\;\mathrm{products}\;\mathrm{are}\;\mathrm{not}\;\mathrm{disassembled}\\ 1,\;\mathrm{when}\;\mathrm{the}\;\mathrm{defective}\;\mathrm{finished}\;\mathrm{products}\;\mathrm{are}\;\mathrm{disassembled} \end{array}\right.,$$

When $D_{3}=0$ occurs, the defective finished products are directly discarded without disassembly, incurring no additional cost.

When $D_{3}=1$ occurs, disassembly is required, and the disassembled spare parts will re-enter the spare part inspection stage. In this case, the cost incurred is solely the disassembly cost, namely:

(17) $$C_{cc}=Q_{c}P_{c}l_{c},$$

where $C_{cc}$ represents the disassembly cost of the defective finished product, and $l_{c}$ denotes the disassembly cost per defective finished product.

To summarize, the total cost at this stage consists of the disassembly cost of the finished products:

(18) $$C_{cc}=D_{3}Q_{c}P_{c}l_{c}.$$

- Replacement Stage for Sold Defective Products

In this stage, the company replaces defective finished products that have already been sold, incurring return costs. The returned defective products will enter the defective product disassembly stage. At this point, the required cost consists of the replacement cost and the return cost of the finished products, namely:

(19) $$C_{cd}+C_{ct}=Q_{c}P_{c}(e_{c}+r_{c}),$$

where $C_{cd}$ represents the replacement cost of the finished products, $C_{ct}$ represents the return cost, $e_{c}$ denotes the replacement cost per finished product, and $r_{c}$ denotes the return cost per finished product. In this paper, the return cost per finished product is defined as the sum of the purchasing costs of the two spare parts and their assembly cost, calculated as follows:

(20) $$r_{c}=u_{1}+u_{2}+a_{c}.$$

- Semi-Finished Product Inspection Stage

Introduce a decision variable $D_{4}$, which represents whether to inspect the semi-finished products, namely:

(21) $$D_{4}=\left\{\begin{array}{l} 0,\;\mathrm{when}\;\mathrm{the}\;\mathrm{semi-finished}\;\mathrm{products}\;\mathrm{are}\;\mathrm{not}\;\mathrm{inspected}\\ 1,\;\mathrm{when}\;\mathrm{the}\;\mathrm{semi-finished}\;\mathrm{products}\;\mathrm{are}\;\mathrm{inspected} \end{array}\right.,$$

When $D_{4}=0$ occurs, no inspection is conducted, and the semi-finished products are directly assembled into finished products. Thus, the cost at this stage includes only the assembly cost of the semi-finished products, namely:

(22) $$C_{bz}=Q_{b}a_{b},$$

where $C_{bz}$ represents the assembly cost of the semi-finished products, $Q_{b}$ denotes the quantity of the *b*-th type of semi-finished product, and $a_{b}$ denotes the assembly cost of the *b*-th type.

When $D_{4}=1$ occurs, defective semi-finished products proceed to the next disassembly stage, while qualified ones are assembled into finished products. Therefore, the cost at this stage includes both the assembly and inspection costs of the semi-finished products, namely:

(23) $$C_{bz}+C_{bj}=Q_{b}(a_{b}+d_{b}),$$

where $C_{bj}$ represents the inspection cost of the semi-finished products, and $d_{b}$ denotes the inspection cost of the *b*-th type of semi-finished product.

To summarize, the total cost at this stage consists of the assembly cost and inspection cost of the semi-finished products:

(24) $$C_{bz}+C_{bj}=Q_{b}a_{b}+D_{4}Q_{b}d_{b}.$$

- Defective Semi-Finished Product Disassembly Stage

Introduce a decision variable $D_{5}$, which represents whether to disassemble defective semi-finished products, namely:

(25) $$D_{5}=\left\{\begin{array}{l} 0,\;\mathrm{when}\;\mathrm{the}\;\mathrm{defective}\;\mathrm{semi-finished}\;\mathrm{products}\;\mathrm{are}\;\mathrm{not}\;\mathrm{disassembled}\\ 1,\;\mathrm{when}\;\mathrm{the}\;\mathrm{defective}\;\mathrm{semi-finished}\;\mathrm{products}\;\mathrm{are}\;\mathrm{disassembled} \end{array}\right.,$$

When $D_{5}=0$ occurs, the defective semi-finished products are directly discarded without incurring additional cost.

When $D_{5}=1$ occurs, disassembly is required. The disassembled spare parts will re-enter the inspection stage, and the cost incurred at this stage is solely the disassembly cost:

(26) $$C_{bc}=Q_{b}P_{b}l_{b},$$

where $C_{bc}$ represents the disassembly cost of the defective semi-finished products, $P_{b}$ denotes the defect rate of the *b*-th type of semi-finished product, and $l_{b}$ is the disassembly cost of the *b*-th type.

To summarize, the total cost at this stage is the disassembly cost of the semi-finished products:

(27) $$C_{bc}=D_{5}Q_{b}P_{b}l_{b}.$$

- Total Cost

To summarize, the objective function is to minimize the total cost across all stages, namely:

(28) $$\min Z=(C_{\mathrm{lg}}+C_{lj})+(C_{bz}+C_{bj})+C_{bc}+(C_{cz}+C_{cj}+C_{cs})+C_{cc}+(C_{cd}+C_{ct}).$$

Constraints:

The quantities of spare parts, semi-finished products, and finished products must all be positive integers, namely:

(29) $$Q_{i},Q_{b},Q_{c}\subseteq N^{+}.$$

Furthermore, it must be ensured that all spare parts are used up during semi-finished product assembly, and all semi-finished products are used up during finished product assembly, namely:

(30) $$Q_{b}\leq \min Q_{i},Q_{c}\leq \min Q_{b}.$$

In conclusion, the decision-making model for multiple processes and multiple parts is as follows:

(31) $$\begin{array}{l} \min Z=(C_{\mathrm{lg}}+C_{lj})+(C_{bz}+C_{bj})+C_{bc}+(C_{cz}+C_{cj}+C_{cs})+C_{cc}+(C_{cd}+C_{ct}).\\ s.t.\left\{\begin{array}{l} Q_{i},Q_{b},Q_{c}\subseteq N^{+}\\ Q_{b}\leq \min Q_{i},Q_{c}\leq \min Q_{b} \end{array}\right. \end{array}$$

### 3.4. Improved Grey Wolf Optimizer (IGWO)

Confronted with the high-dimensional and non-linear optimization landscape shaped by uncertain defect rates, traditional solvers risk converging on locally optimal yet globally inefficient production policies. To overcome this challenge, the MsPIO model leverages the Improved Grey Wolf Optimizer (IGWO), whose pack-based cooperative search mechanism is inherently suited to the characteristics of Uncertain Multi-stage Production. By simulating hierarchical leadership and collaborative hunting behavior, IGWO deploys multiple search agents to simultaneously explore and exploit diverse regions of the solution space, thereby reducing the risk of premature convergence and ensuring a robust global search. Its suitability is further reinforced under our assumptions: (i) the independence of sample qualification allows IGWO’s parallel agents to evaluate production stages without correlation bias; (ii) the accuracy of the enterprise’s inspection system ensures that optimization outcomes are not distorted by measurement errors; and (iii) the guarantee that all finished products are successfully sold aligns the objective function directly with profit maximization. Prior evidence has shown that IGWO performs effectively in complex, stochastic environments, and its adaptive exploration–exploitation balance makes it particularly well matched to the uncertainty and asymmetry inherent in multi-stage production systems.

In this work, an Improved Grey Wolf Optimizer (IGWO) is proposed to address the MsPIO problem. The conventional GWO [41], which draws inspiration from the social hierarchy and hunting behavior of grey wolves, is enhanced in the following three aspects. First, Latin Hypercube Sampling (LHS) is employed to generate the initial population, significantly improving its spatial uniformity. Second, a simulated binary crossover (SBX)-based evolution operator is introduced within the triple-parent framework to produce potential candidate solutions, thereby strengthening the algorithm’s exploration capability in complex search spaces. Finally, a mutation-based opposition learning mechanism is incorporated, which generates opposite solutions for inferior individuals and selectively replaces them to mitigate premature convergence.

#### 3.4.1. GWO Original Position Update Steps

1. Encircling the Prey

Firstly, the grey wolves’ ranks need to be classified, with the top-level wolves referred to as leader wolves (the optimal solution), represented by α, β, and δ. These leader wolves guide the rest of the wolf pack to hunt the prey. The other wolves (candidate solutions) are denoted as ω, and they locate and determine the target based on the leader wolves’ instructions. During the hunting process, the behavior of the grey wolves encircling the prey is described as follows:

(32) $$\vec{D}=\left|\vec{C}{\vec{X}}_{P}(t)-\vec{X}(t)\right|,$$

(33) $$\vec{X}(t+1)={\vec{X}}_{P}(t)-\vec{A}\vec{D},$$

where $\vec{D}$ represents the distance between the grey wolf and the prey, $\vec{X}(t+1)$ is the updated position of the grey wolf, *t* is the current iteration number, $\vec{A}$ and $\vec{C}$ are coefficient vectors, ${\vec{X}}_{P}$ represents the position vector of the prey, and $\vec{X}$ denotes the position vector of the grey wolf.

The following are the formulas for the coefficient vectors:

(34) $$\vec{A}=2\vec{a}{\vec{r}}_{1}-\vec{a},$$

(35) $$\vec{C}=2{\vec{r}}_{2},$$

where $\vec{a}\;\;$ is the convergence factor, and as the iteration number progresses, it decreases from 2 to 0. ${\vec{r}}_{1}$ and ${\vec{r}}_{2}\;\;$ are random numbers between [0, 1].

2. Hunting the Prey

**Figure 2:**
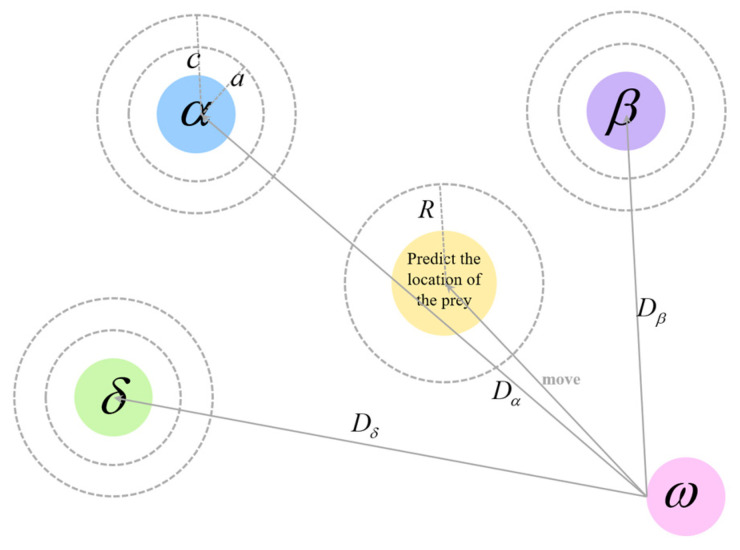
Grey Wolf Individual Position Update Diagram.

Once the leader wolves identify the prey’s location, they guide the pack to gradually encircle and hunt it. In the optimization decision process, the exact position of the prey is unknown. Therefore, to simulate the grey wolves’ hunting behavior, it is assumed that α, β, and δ have a clearer estimation of the prey’s potential location. These three wolves are considered the best solutions, and their positions are used to search for the possible location of the prey. Meanwhile, the rest of the grey wolves adjust their positions based on the leaders’ locations, gradually approaching the prey. This strategy helps get closer to the prey’s actual position, thereby improving solution quality. Figure 2 displays the update process of an individual wolf’s position.

Accordingly, the grey wolf hunting model is established as follows:

(36) $$\left\{\begin{array}{l} {\vec{D}}_{\alpha }=\left|{\vec{C}}_{1}{\vec{X}}_{\alpha }-\vec{X}\right|\\ {\vec{D}}_{\beta }=\left|{\vec{C}}_{2}{\vec{X}}_{\beta }-\vec{X}\right|\\ {\vec{D}}_{\delta }=\left|{\vec{C}}_{3}{\vec{X}}_{\delta }-\vec{X}\right| \end{array}\right.,$$

where the distances between the leader wolves and other individuals are represented by ${\vec{D}}_{\alpha }$, ${\vec{D}}_{\beta }$, and ${\vec{D}}_{\delta }$, the random vectors are denoted by ${\vec{C}}_{1}$, ${\vec{C}}_{2}$, and ${\vec{C}}_{3}$, and the current positions of the leader wolves are represented by ${\vec{X}}_{\alpha }$, ${\vec{X}}_{\beta }$, and ${\vec{X}}_{\delta }$, respectively.

After further rearranging the formula, we obtain the step size and direction of the rest of the wolves as they move toward the leader wolves, given by:

(37) $$\left\{\begin{array}{l} {\vec{X}}_{1}={\vec{X}}_{\alpha }-A_{1}{\vec{D}}_{\alpha }\\ {\vec{X}}_{2}={\vec{X}}_{\beta }-A_{2}{\vec{D}}_{\beta }\\ {\vec{X}}_{3}={\vec{X}}_{\delta }-A_{3}{\vec{D}}_{\delta } \end{array}\right..$$

Finally, the position update for the remaining grey wolves ω in the population is as follows:

(38) $$\vec{X}(t+1)=\frac{{\vec{X}}_{1}+{\vec{X}}_{2}+{\vec{X}}_{3}}{3}.$$

3. Besieging the Prey

When the prey stops moving, the wolf pack can complete the hunt by surrounding and besieging it. During the simulation of approaching the prey, the value of $\vec{a}$ gradually decreases, which in turn narrows the range of changes for $\vec{A}$. Specifically, as $\vec{a}$ decreases from 2 to 0, the value of $\vec{A}$ will fluctuate within the range of $\left[-a,a\right]$.

#### 3.4.2. Improvement Strategies

1. LHS Initialization

Latin Hypercube Sampling (LHS) [42] is an efficient initialization method based on stratified sampling that ensures a uniform distribution of sample points across a multidimensional search space. In the hunting process of grey wolves, individuals are typically dispersed uniformly across the terrain to conduct extensive exploration, thereby increasing the probability of locating prey. Inspired by this behavioral characteristic, this study integrates LHS into the algorithmic framework to mitigate the non-uniformity of samples caused by random initialization, while simultaneously enhancing global search capability.

Assume the search space is *D*-dimensional, and the value range in each dimension is the interval $\left[x_{\min}^{i},x_{\max}^{i}\right]$(i=1,2,⋯,D). For each dimension *i*, the range is partitioned into *N* equiprobable subintervals. The length of each subinterval is:

(39) $$\Delta x^{i}=\frac{x_{\max}^{i}-x_{\min}^{i}}{N}.$$

A uniformly distributed random value $r_{ij}\in \left[0,1\right]$ is selected within the *j*-th subinterval of the *i*-th dimension. A sample point is then positioned within this subinterval based on this random value:

(40) $$x_{ij}=x_{\min}^{i}+(j-1+r_{ij})\Delta x^{i}.$$

To ensure a uniform distribution of sample points across the entire search space, a random permutation is applied to the sampling order across different dimensions, guaranteeing that the samples are evenly distributed in all dimensions. Subsequently, an initial population of *N* individuals is constructed, where the value of each dimension for every individual is systematically drawn from the corresponding subinterval using the LHS method:

(41) $$x_{j}=[x_{1i},x_{2j},\cdots ,x_{Dj}],j=1,2,\cdots ,N.$$

2. Evolutionary Parent Roundup

In nature, grey wolves also achieve efficient hunting through group cooperation and dynamic adjustment strategies, which are typically characterized by diverse hunting behaviors. Building upon this concept, this study incorporates Simulated Binary Crossover (SBX) [43] into the IGWO framework. As a crossover operator that simulates the behavior of binary crossover in genetic algorithms, SBX effectively introduces evolutionary variation by leveraging three-parent solutions to generate new offspring. This strategy enhances the diversity and complexity of parental information, enabling the algorithm to more effectively escape local optima and accelerate the search for the global optimum.

SBX determines the inheritance of genetic information between offspring based on the differences between the two parent solutions. The crossover factor χ is first calculated using SBX as follows:

(42) $$\chi =\left\{\begin{array}{l} {\left(2u\right)}^{\frac{1}{\eta_{c}+1}},\;\;\;\;\;\;\;\;\;\;\;\;\;\;u\leq \frac{1}{2}\\ {\left(\frac{1}{2(1-u)}\right)}^{\frac{1}{\eta_{c}+1}},u>\frac{1}{2} \end{array}\right.,$$

where u is a random number and $\eta_{c}=2$ is the crossover distribution index controlling the shape of the crossover operator. The three-parent strategy further incorporates the relative positional differences among individuals α, β, and δ. By systematically combining their characteristics, this approach explores new regions of the solution space, thereby effectively leveraging information from high-quality solutions to generate improved offspring. The evolutionary factor (EF) is computed as follows:

(43) $$EF=\frac{\beta (\alpha_{pos}^{j}-\beta_{pos}^{j})+(1-\beta )(\beta_{pos}^{j}-\delta_{pos}^{j})}{2},$$

Finally, the wolf’s position is updated:

(44) $$x_{ij}^{new}=\frac{EF+X_{1}+X_{2}+X_{3}}{4},$$

Additionally, we ensure that each wolf’s new position does not exceed the defined search space boundaries:

(45) $$X_{ij}=\max(\min(X_{ij},ub_{j}),lb_{j}),$$

At the same time, compare the fitness values of the new position and the old position. If the fitness of the new position is better, replace the old position:

(46) $$if\;\;\;f_{new}<f_{old},X_{i}^{old}=X_{i}^{new}.$$

3. Mutation Reverse Learning Strategy

When facing hunting failures or encountering more resilient prey, grey wolves often exhibit strategic adaptability by altering their attack patterns or reorganizing their formation to reinitiate the hunt, all while refining the capabilities of underperforming members to strengthen the overall group. Inspired by this observed behavior, this study incorporates a mutation-based opposition learning strategy [44]. This approach enhances population diversity by applying mutation operations to the least fit individuals, enabling the IGWO to autonomously adjust and escape local optima when trapped in suboptimal regions, thereby enhancing its global search capability.

Specifically, individuals are ranked in descending order based on their fitness values, and the lowest-performing quartile of individuals is selected for opposition-based learning:

(47) $$worst_{indices}=sort(f_{values^{\prime },descend^{\prime }})(1:\frac{N}{4}),$$

Calculate the inverse solution of α, β, and δ:

(48) $$X_{\alpha }^{\prime }=lb+ub-\alpha_{pos},$$

(49) $$X_{\beta }^{\prime }=lb+ub-\beta_{pos},$$

(50) $$X_{\delta }^{\prime }=lb+ub-\delta_{pos},$$

Calculate the reverse solution for the worst individual $X_{worst}$ and add the Gaussian perturbation term ε:

(51) $$X^{\prime }=lb+ub-X_{worst}+\varepsilon ,$$

where $\varepsilon =(ub_{j}-lb_{j})randn$. In addition, calculate the fitness value of the reverse solution:

(52) $$f_{opposition}=\mathrm{Cos}tFunction(X^{\prime }),$$

Then select the optimal reverse solution according to the fitness value:

(53) $$best_{oppositions}=sort(oppositions_{fitness^{\prime },ascend^{\prime }})(1:\frac{N}{4}),$$

Similarly, if the fitness of the reverse solution is better, replace the original individual:

(54) $$if\;\;\;f_{best\;opposition}<f,X_{worst}=X_{best\;opposition},$$

And update the positions and scores of α, β, and δ:

(55) $$if\;\;\;f_{best\;opposition}<\alpha_{score},\alpha_{pos}=X_{best\;opposition},\alpha_{score}=f_{best\;opposition},$$

(56) $$if\;\;\;\alpha_{score}<f_{best\;opposition}<\beta_{score},\beta_{pos}=X_{best\;opposition},\beta_{score}=f_{best\;opposition},$$

(57) $$if\;\;\beta_{score}<f_{best\;opposition}<\delta_{score},\delta_{pos}=X_{best\;opposition},\delta_{score}=f_{best\;opposition}.$$

**Figure 3:**
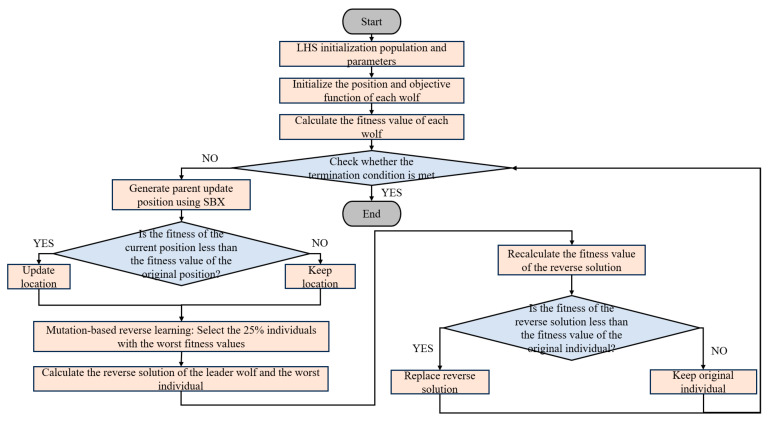
IGWO Flowchart.

The complete algorithm flow chart is shown in Figure 3, where, orange represents flowcharts, and blue represents decision boxes.

**Figure 4:**
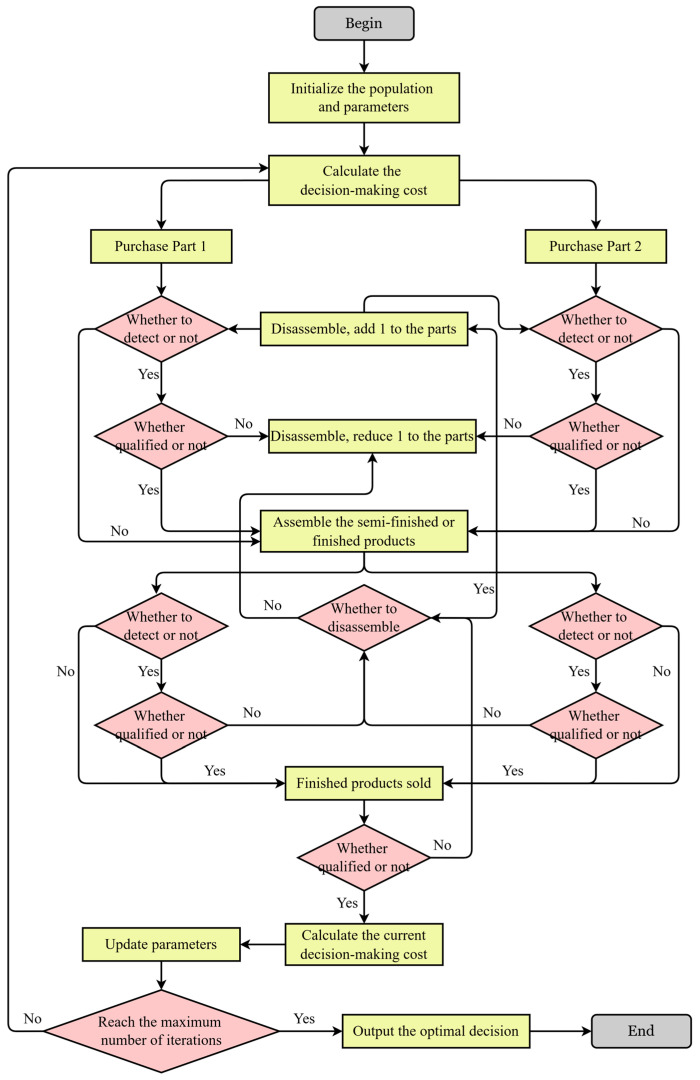
IGWO Solution Flowchart.

The complete solution flowchart is shown in Figure 4, and the specific steps are as follows:

**Step 1.** Initialize the Wolf Pack: Based on the problem scale and constraints, initialize a wolf pack. Each wolf represents a decision scheme.

**Step 2.** Initial Strategy Cost: For each wolf’s decision scheme, calculate its corresponding total cost.

**Step 3.** Decide Whether to Inspect Spare Parts: Each wolf decides whether to inspect spare parts. If inspected, the purchased spare parts are checked for quality. If they pass, they move on to the qualification check; if they do not pass, they are discarded. If not inspected, the spare parts are directly assembled.

**Step 4.** Decide Whether to Inspect Assembled Products: Each wolf decides whether to inspect the assembled finished or semi-finished products. If inspected and passed, they move on to the qualification check; if not, proceed to Step 5. If not inspected, the products are directly sold.

**Step 5.** Decide Whether to Disassemble Defective Finished Products: Each wolf decides whether to disassemble defective finished or semi-finished products. If disassembled, the spare parts are retrieved, and the process returns to Step 3. Otherwise, the product is discarded. Even uninspected defective products sold will reach this step.

**Step 6.** Update Position: Update the wolf’s position, i.e., the optimal decision, and calculate the cost.

**Step 7.** Fitness Evaluation: Evaluate the cost of each decision scheme and select the one with the lowest cost to update the current decision.

**Step 8.** Iterative Optimization: Repeat the above process until the predetermined number of iterations is reached or the convergence condition is met. Output the decision scheme with the minimum cost.

## 4. Experimental Analysis

### 4.1. A Case Study

In this experiment, we used examples from the “China Mathematical Contest in Modeling”. This case includes both single-process and multi-process production scenarios. Appendix A displays the defect rates and prices for various spare parts in a single-process scenario, where the process involves assembling two spare parts into one finished product.

**Figure 5:**
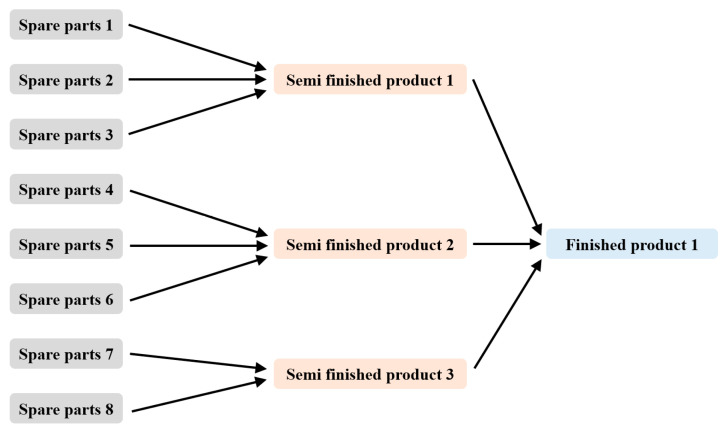
Multi-process Assembly Scenario.

Figure 5 illustrates the multi-process scenario, where two or three components are assembled into a semi-finished product, and then three semi-finished products are assembled into a finished product. In this diagram, light gray boxes represent individual spare parts, the basic elements of production. Light orange boxes represent semi-finished products assembled from several spare parts. Light blue boxes represent the finished product after final assembly. Black arrows indicate the assembly or material flow, i.e., the process path of how spare parts are combined into semi-finished products, and how semi-finished products are then combined into finished products. Appendix A presents the defect rates and prices for various components in the multi-process scenario.

### 4.2. Inspection Results

Based on the two-stage sampling inspection model in the rejection plan, let α=0.05 be set, with the total number of components *N* = 500 and the threshold for defective parts $D_{0}=50$. The result shows that the minimum sample size is *n* = 2, and the rejection critical number is *c* = 2. This means that if two components are selected from the 500 components and both are defective (*X* = 2), the rejection decision is made with 95% confidence. Otherwise (*X* = 0 or 1), the rejection cannot be made. In the acceptance plan, let β=0.1 be set with the total number of components *N* = 500 and the threshold for defective parts $D_{0}=50$. The result shows that the minimum sample size is *n* = 22, and the acceptance critical number is *m* = 0. This means that if 22 components are selected and all are non-defective (*X* = 0), the acceptance decision is made with 90% confidence. Otherwise (*X* ≥ 1), the acceptance cannot be made.

Based on the two schemes above, the two-stage sequential sampling plan is implemented as follows: The first stage selects a sample size of $n_{1}=2$. If the number of defects $X_{1}\geq 2$, the batch is immediately rejected; otherwise, the process proceeds to the second stage. The second stage involves an additional sample of size $n_{2}=20$, making the total sample size 22. The batch is accepted only if the total number of defects X≤0; otherwise, reject it. After 10,000 simulation trials, the batch was accepted in 986 cases, resulting in an acceptance rate of 9.86%, and rejected in 9014 cases, giving a rejection rate of 90.14%.

Subsequently, the Clopper–Pearson method was applied with a 95% confidence level to estimate the defect rate for accepted batches. The estimated defect rate for the entire batch of components in such cases falls within the interval [0, 0.154].

The following outlines the derivation process for the defect-rate intervals of semi-finished products and finished products for two components. Let the fixed defect rate in the assembly process be $P_{z}=0.1$, the probability that both components are qualified be $\left(1-P_{l1})(1-P_{l2}\right)$, and the probability of at least one defective component be $1-(1-P_{l1})(1-P_{l2})$. The condition for a semi-finished product to be qualified is that all components are qualified and the assembly is correct. Therefore, the semi-finished product pass rate is $\left(1-P_{z})(1-P_{l1})(1-P_{l2}\right)$, and the semi-finished product defect rate $P_{b}$ is:

(58) $$P_{b}=1-(1-P_{z})(1-P_{l1})(1-P_{l2}),$$

where $P_{l1}$ and $P_{l2}$ are the defect rates of the two spare parts.

Similarly, the defect rate $P_{c}$ of finished products can be obtained as:

(59) $$P_{c}=1-(1-P_{z})(1-P_{b1})(1-P_{b2})(1-P_{b3}),$$

where $P_{b1}$, $P_{b2}$, and $P_{b3}$ are the defect rates of the three semi-finished products.

In summary, the defect rates of semi-finished and finished products for various production scenarios are summarized in Table 2.

### 4.3. Experimental Results Analysis

This section employs the commercial optimization-solving software MATLAB R2022b on a computer with an 11th-generation Intel(R) i7 CPU, using the parameters provided in the previous chapter for model resolution.

#### 4.3.1. Performance Testing

To evaluate the performance of our proposed IGWO, we conducted comparative experiments against two recently enhanced grey wolf optimizer variants: the hybrid grey wolf and whale optimization algorithm (hGWOA) [45] and the method integrating random opposition-based learning, strengthened wolf hierarchy, and modified evolutionary population dynamics (RSMGWO) [46]. The algorithms were tested on the IEEE CEC2022 benchmark suite [47], a comprehensive set of optimization problems detailed in Table 3.

To ensure experimental reproducibility, the initial population size for each intelligent algorithm is mandated to be 50, with a maximum of 1000 iterations permitted. Each algorithm undergoes 50 independent experimental trials, validated on the IEEE CEC 2022 benchmark suite (10-dimensional space).

Before the test began, this study set fixed initial parameters for the algorithm as shown in Table 4.

Firstly, ablation experiments were conducted to test the performance of each element in the proposed IGWO. GWO-LHS represents GWO initialized with Latin Hypercube Sampling, GWO-SBX represents GWO improved with Simulated Binary Crossover mechanism, and GWO-OL represents GWO enhanced with opposition learning strategy.

Table 5 and the corresponding figure unequivocally demonstrate the individual and synergistic contributions of each proposed improvement to the final IGWO algorithm. On the most challenging functions, the complete IGWO achieves superior accuracy and convergence. For instance, on F1, IGWO’s average (3.40 × 10^2^) significantly outperforms not only the basic GWO (1.68 × 10^3^) but also all its intermediate variants, including GWO-SBX (3.54 × 10^2^) and GWO-OL (4.17 × 10^2^), highlighting that the fusion of all components is crucial for optimal performance. A similar trend is observed on F6 and F11, where IGWO attains the best average values (5.35 × 10^3^ and 2.70 × 10^3^, respectively), underscoring its enhanced capability in navigating complex, high-dimensional search spaces. Furthermore, the consistently lower standard deviation of IGWO across a majority of functions (e.g., F1, F4, F9, F10, F11) confirms its superior stability and reliability compared to the other configurations. This combination of the lowest average fitness and minimal performance variability makes a compelling case for the robustness of the fully assembled IGWO algorithm, proving that each component—LHS, SBX, and OL—plays a vital and complementary role in the overall design.

**Figure 6:**
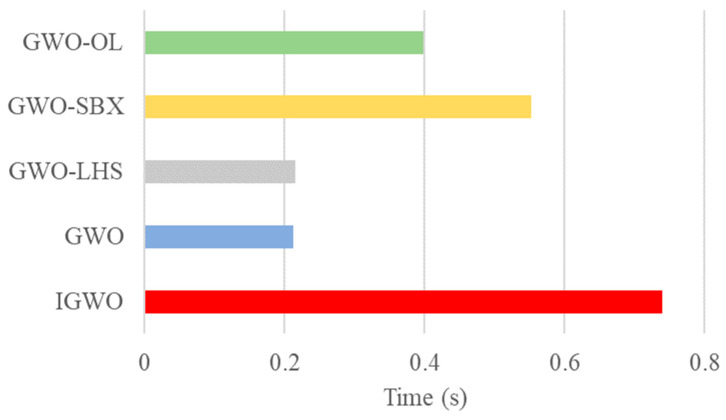
Running Time of Each Element.

Figure 6 depicts the computational time consumption of the proposed algorithm and its variants, where the standard GWO algorithm serves as the computational baseline. The results indicate that the IGWO algorithm incurs a substantial computational overhead, requiring approximately 3.5 times longer to execute than the baseline GWO. This is a direct consequence of integrating the additional LHS, SBX, and OL mechanisms. Among the individual components, the GWO-SBX variant exhibits the most significant time increase, implying that the crossover operation is the primary contributor to the computational cost. Meanwhile, the GWO-LHS variant shows a negligible time increase compared to the baseline, confirming the efficiency of the initialization strategy. This notable rise in runtime for the complete IGWO is, however, a justified trade-off for its demonstrably superior convergence accuracy and robust performance, as comprehensively validated in our experimental sections.

Table 6 presents the *p*-values derived from the Wilcoxon signed-rank test, which is employed to statistically ascertain the performance differences between the proposed IGWO and its component variants. The IGWO algorithm demonstrates statistically significant superiority over the base GWO, as well as the GWO-LHS and GWO-SBX variants. This is evidenced by the exceptionally low *p*-values in its corresponding row: 2.44 × 10^−3^ against GWO, 4.88 × 10^−3^ against GWO-LHS, and 2.69 × 10^−2^ against GWO-SBX. These results strongly reject the null hypothesis of equivalent performance, confirming that the integration of all three components in IGWO yields a performance that is significantly better than that of the algorithm with only one improvement. Notably, the *p*-value of 0.470 against GWO-OL indicates that their performance is not statistically different, highlighting the substantial individual contribution of the opposition learning strategy. In conclusion, while the OL strategy alone confers a performance level comparable to the full IGWO, the complete algorithm robustly outperforms the baseline and other intermediate forms, solidifying its overall efficacy.

**Figure 7:**
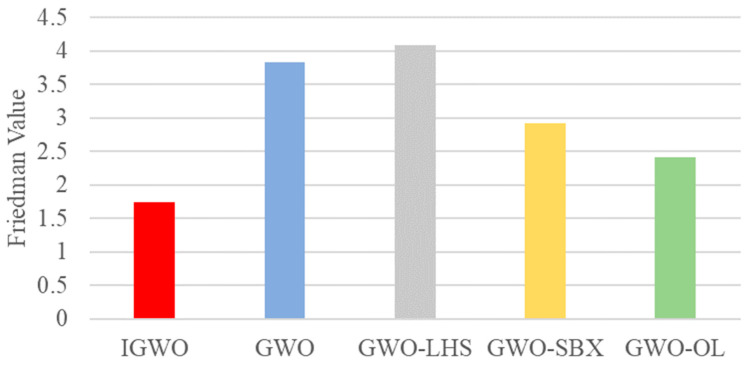
The Friedman Value of Each Element on the CEC2022 Test Suite (Dim = 10).

Figure 7 summarizes the Friedman test results, which provide an overall performance ranking of the algorithms. The proposed IGWO algorithm secures the top ranking with the lowest Friedman value of 1.75, unequivocally identifying it as the best-performing method. The GWO-OL variant achieves the second position with a value of 2.42, followed by GWO-SBX at 2.92. In contrast, the base GWO and GWO-LHS obtain the highest (i.e., worst) Friedman values of 3.83 and 4.08, respectively, confirming their inferior performance relative to the enhanced variants in this study. This statistical ranking definitively positions the complete IGWO as the most effective algorithm, demonstrating that the synergistic integration of all components yields superior overall performance compared to any individual improvement.

After that, to test the advantages of the proposed IGWO over the previous GWO variant, the CEC 2022 kit (Dim = 10) was also used.

**Figure 8:**
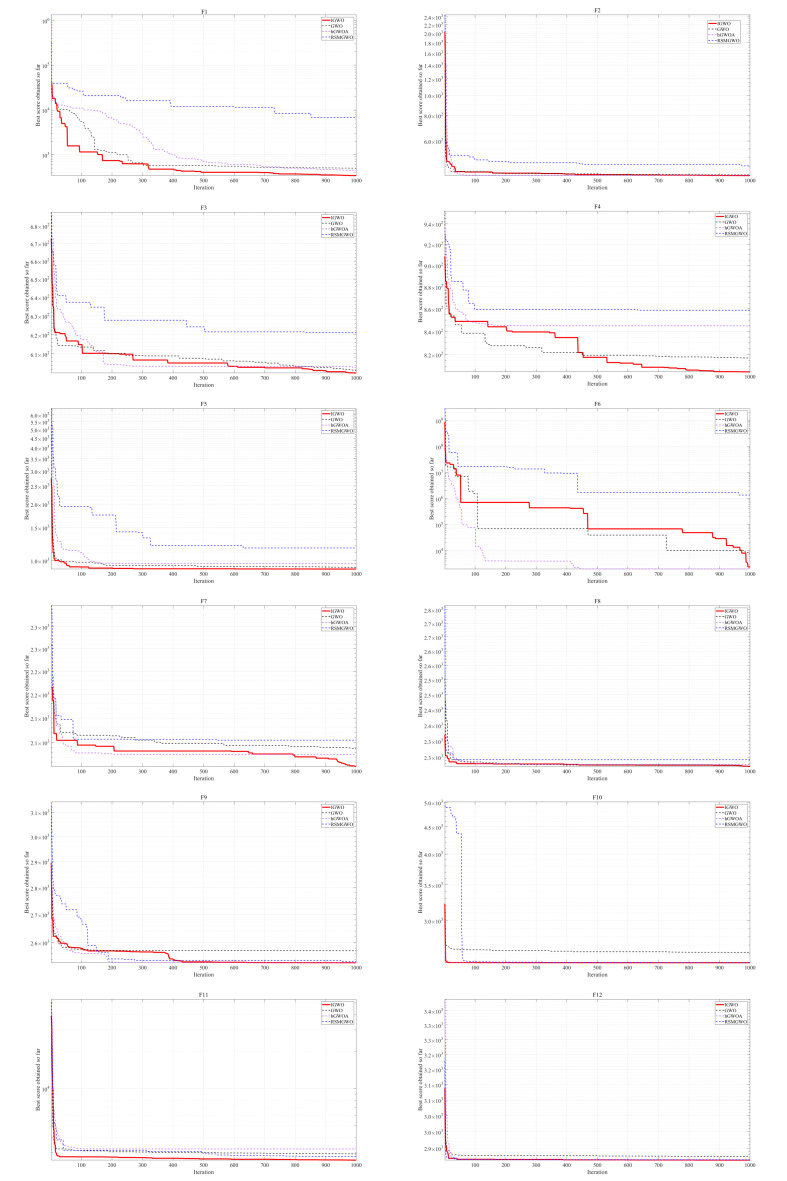
The Convergence Curves of Different Algorithms.

Table 7 and Figure 8 indicate that on complex functions such as F1 and F6, IGWO achieves far superior average values (3.48 × 10^2^ and 4.73 × 10^3^, respectively) compared to its competitors, and IGWO has a fast convergence speed. For instance, on F1, IGWO’s average is an order of magnitude better than that of GWO (1.22 × 10^3^) and dramatically outperforms RSMGWO (4.91 × 10^3^). This indicates a significantly enhanced capability for escaping local optima and locating the vicinity of the global optimum. Furthermore, the notably lower standard deviation values of IGWO across most functions (e.g., F1, F5, F6, F11) underscore its superior stability and reliability. A smaller Std signifies that IGWO’s performance is less variable across independent runs, yielding highly consistent and dependable results. This combination of low Avg and low Std makes a compelling case for the robustness of the proposed algorithm.

**Figure 9:**
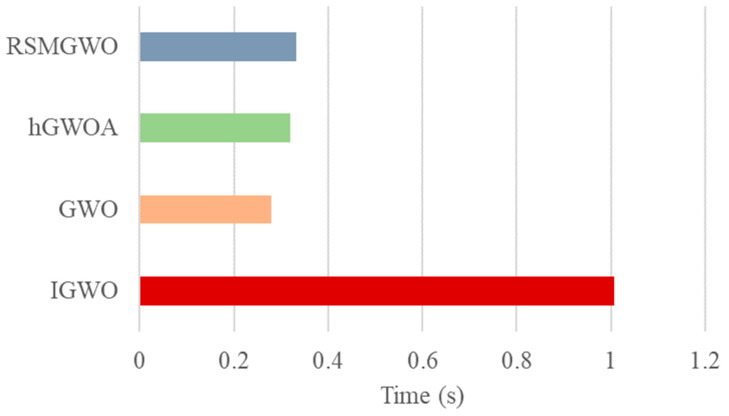
Running Time of Different Algorithms.

Figure 9 shows that the standard GWO algorithm serves as the computational baseline, with IGWO, hGWOA and RSMGWO exhibiting marginally longer execution periods. Notably, the IGWO algorithm incurs a substantial computational overhead, requiring approximately 3.6 times longer to execute than the baseline GWO. This significant increase in runtime, however, is a direct and justified trade-off for its demonstrably superior convergence properties and markedly enhanced solution precision observed in our experimental validation.

Table 8 presents the *p*-values derived from the Wilcoxon signed-rank test, which is employed to statistically ascertain the performance differences between the various algorithms. The proposed IGWO algorithm demonstrates statistically significant superiority over all its competitors. This is evidenced by the exceptionally low *p*-values (all below 0.05) in its corresponding row: 2.44 × 10^−3^ against GWO, 2.69 × 10^−2^ against hGWOA, and 1.47 × 10^−3^ against RSMGWO. These results strongly reject the null hypothesis of equivalent performance, confirming that IGWO’s enhanced convergence accuracy, as previously noted, is not incidental but statistically robust. In conclusion, IGWO is the top-performing algorithm, achieving a level of solution quality that is significantly better than that of the other state-of-the-art methods tested.

**Figure 10:**
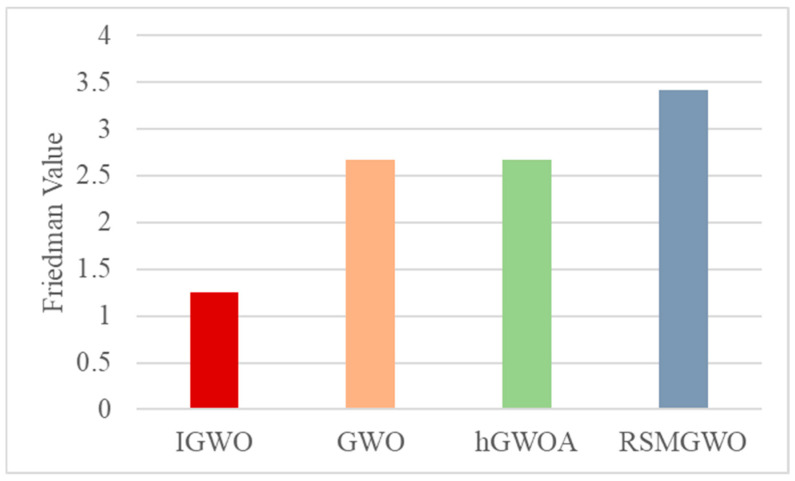
The Friedman Value of Different Algorithms on the CEC2022 Test Suite (Dim = 10).

Figure 10 shows that ESFOA exhibits the best performance on the F7, F8, F10, and F12. Figure 8 shows the proposed IGWO algorithm secures the top ranking with the lowest Friedman value of 1.25, unequivocally identifying it as the best-performing method overall. The algorithms GWO and hGWOA are tied for the second position, both with an average rank of 2.67. Finally, the RSMGWO algorithm obtains the highest (i.e., worst) Friedman value of 3.42, indicating its inferior performance relative to the other algorithms in this comparative study. It definitively ranks IGWO as the most effective algorithm.

Finally, IGWO was tested on the CEC 2022 kit (Dim = 10) and compared with global optimization methods, differential algorithms (DE), and the Covariance Matrix Adaptation Evolution Strategy (CMA-ES) to verify the powerful global optimization capabilities of IGWO.

Table 9 presents a comparative performance analysis of the proposed IGWO against two mainstream global optimizers, DE and CMA-ES, on the CEC 2022 benchmark suite. The results demonstrate that IGWO achieves highly competitive, and often superior, performance. On complex unimodal and hybrid functions, IGWO exhibits a decisive advantage. For instance, on F1, IGWO’s average value (3.40 × 10^2^) is dramatically lower than that of DE (1.59 × 10^3^) and CMA-ES (9.50 × 10^2^), highlighting its superior capability for exploitation and convergence to high-precision solutions. A similar trend is observed on F11, where IGWO’s average (2.70 × 10^3^) significantly outperforms both DE (3.11 × 10^3^) and CMA-ES (2.87 × 10^3^). Furthermore, while DE shows strong performance on functions like F6 and F7, IGWO maintains robust competitiveness across the majority of test functions. The consistently low standard deviation of IGWO on key functions such as F1, F4, and F11 further underscores its stability and reliability. This comprehensive comparison confirms that the proposed IGWO is not only an improvement over its GWO-based peers but also a formidable contender against established mainstream optimizers, successfully balancing exploratory power with convergent precision.

**Figure 11:**
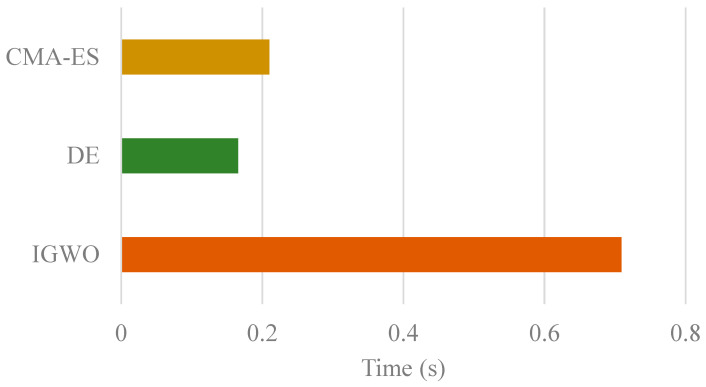
Running Time of Different Global Optimization Methods.

The computational time consumption of IGWO and the mainstream optimizers is presented in the accompanying Figure 11. The results indicate that the proposed IGWO algorithm incurs a substantial computational overhead compared to the highly efficient DE and CMA-ES algorithms. Specifically, IGWO requires approximately 4.3 times longer to execute than DE and 3.4 times longer than CMA-ES. This significant increase in runtime is a direct consequence of the sophisticated integration of the LHS initialization, SBX, and opposition-based learning mechanisms within the IGWO framework. While DE demonstrates the fastest execution, this notable rise in runtime for the complete IGWO is a justified and necessary trade-off for its demonstrably superior convergence accuracy and robust performance across the CEC 2022 benchmark, as conclusively validated in the preceding sections.

Table 10 presents the *p*-values from the Wilcoxon signed-rank test conducted to statistically ascertain the performance differences between IGWO and the mainstream optimizers, DE and CMA-ES. The results indicate that there is no statistically significant difference in the overall performance among the three algorithms. This conclusion is supported by the obtained *p*-values, all of which are substantially above the 0.05 significance threshold: 0.424 between IGWO and DE, 0.151 between IGWO and CMA-ES, and 0.470 between DE and CMA-ES. These high *p*-values fail to reject the null hypothesis, meaning that the observed performance advantages of IGWO in the previous tables, while evident in the average values, are not statistically conclusive at this confidence level. Therefore, it can be stated that the proposed IGWO achieves performance that is statistically competitive with both the classic DE and the state-of-the-art CMA-ES, establishing it as a viable and robust alternative within the landscape of global optimization algorithms.

**Figure 12:**
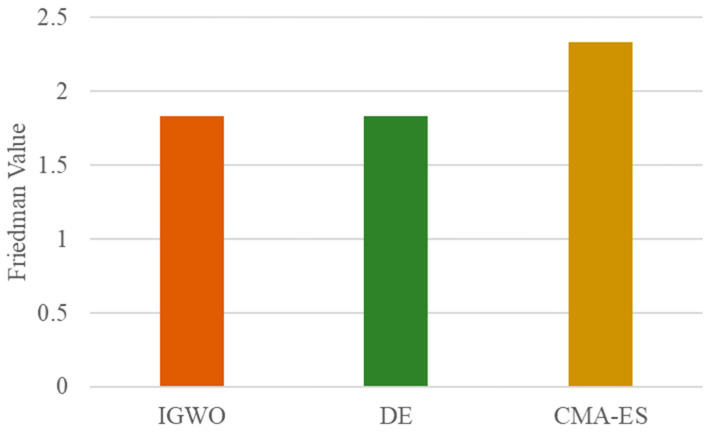
The Friedman Value of Different Global Optimization Methods on the CEC2022 Test Suite (Dim = 10).

The overall performance ranking derived from the Friedman test is presented in Figure 12. The results show that the proposed IGWO algorithm secures the top rank, achieving a top-tier Friedman value of 1.8333. It is noteworthy that IGWO attains the same top rank value as the classic Differential Evolution (DE) algorithm, indicating that their overall performances are statistically equivalent and both are the best-performing methods in this study. The CMA-ES algorithm obtains a slightly higher (i.e., worse) Friedman value of 2.3333. This ranking definitively positions the proposed IGWO as a leading optimizer, whose overall performance is not only superior to the advanced CMA-ES but is also statistically indistinguishable from the highly regarded DE algorithm, thereby solidifying its competitiveness and effectiveness.

#### 4.3.2. Single-Process Test

To begin with, a single-process experimental test was conducted on the model. The model was tested across six different scenarios for the single process. After running the test 100 times, the average decision value was calculated, and the results are shown in Table 11. Please note that all cost units in this article are monetary units (m.u.).

Table 11 demonstrates that the results obtained by our model, with the goal of minimizing costs, consistently yield positive profits across all cases. In Cases 1 to 5, the model determined that inspecting finished products and disassembling unqualified products are essential, with rates fixed at 100%, indicating that these steps are critical for quality assurance despite potential high defect rates or costs. However, in Case 6, the inspection rates for finished products and disassembly are significantly lower (12.16% and 18.52%, respectively), yet the profit is the highest (12,026), suggesting that the model optimized the inspection process by reducing unnecessary checks while maintaining cost efficiency. The varying inspection rates for spare parts (e.g., low rates in Case 1 and high rates in Case 4) reflect adaptive decisions based on defect risks or cost-balancing strategies. Overall, it is evident that our model effectively finds an optimal balance between cost and detection rate in single-process tasks, ensuring profitability while managing quality control.

**Figure 13:**
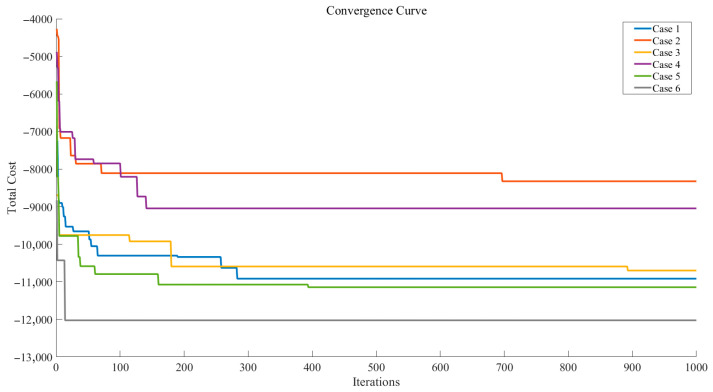
Convergence Curves for Various Situations in a Single Process.

Figure 13 demonstrates that the IGWO quickly raises the total profit to the range of profit of 9000 to 12,000 during the first 200 iterations, after which it fine-tunes to a stable value. This indicates that IGWO is effectively integrated into the solution process of our model.

#### 4.3.3. Multi-Process Test

Subsequently, a multi-process experiment was conducted. A comparison with the similarly improved GWO verifies the effectiveness of the proposed method.

The comparison of multi-process optimization results in Table 12 shows that our IGWO model significantly outperforms the other comparison algorithms with a total profit of 43,800, thanks to its more intelligent and balanced decision-making in many inspection and disassembly links. Compared with hGWOA, which shows a tendency to “over-inspect”, and RSMGWO, whose decision-making has extreme risks, the IGWO model can more finely balance the costs and risks of each link. While ensuring the common bottom line of 100% full inspection of the final product, it minimizes the total cost and maximizes the profit by optimizing the inspection intensity of the early processes, demonstrating its excellent cost control and decision-making robustness in a complex multi-process environment.

**Figure 14:**
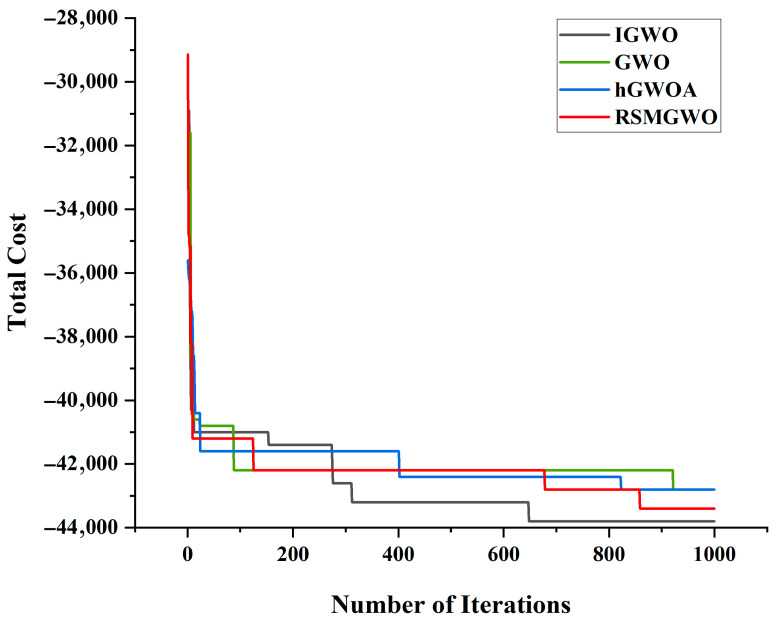
Convergence Curves for Situations in a Multi-process.

As shown in Figure 14, IGWO surpasses GWO and its variants in around 300 iterations. This demonstrates IGWO’s ability to meticulously identify near-optimal detection–decomposition combinations. Through subsequent fine-tuning, the algorithm further reduces costs, highlighting its superior efficiency and robustness.

#### 4.3.4. Confidence Level Analysis

To further validate the decision-making capability of the model under uncertainty, a sensitivity analysis is conducted. First, the uncertainty degree of the confidence level in the two-stage sampling inspection model presented in this paper is analyzed, discussing the impact of different confidence levels on the results under uncertain conditions. The probability estimation results with adjusted confidence levels are shown in Table 13. Similarly, after running the model 100 times, the results are presented in Table 14.

A lower confidence level signifies greater uncertainty in parameter estimates. As illustrated in Table 13, when the confidence level is reduced, the corresponding interval must be situated closer to zero to satisfy the batch acceptance criteria, thereby enabling a more definitive assessment of part quality. Table 14 further reveals that although a 15% confidence level may lead to higher theoretical profits, the associated increase in parameter uncertainty necessitates more extensive inspection efforts. In practical manufacturing settings, estimation outcomes are often suboptimal under such conditions. Thus, it is critical to strategically set the confidence level amid uncertainty, enhance process control measures, and streamline procedures for low-risk components to improve overall profitability.

#### 4.3.5. Finished Product Selling Price Analysis

In addition, the selling price of finished products can fluctuate with market conditions. Therefore, we conducted an uncertainty analysis on the selling price of finished products. Considering varying product prices, this paper will discuss the corresponding outcomes for each price level. The results, after running the model 100 times, are presented in Table 15.

As evidenced by the sensitivity analysis in Table 15, rising finished product prices are accompanied by a marked upward trajectory in total profit, reflecting a strong positive correlation between pricing strategy and overall economic return. Concurrently, the decision-making structure exhibits notable variability across inspection and dismantling activities, with certain processes demonstrating pronounced sensitivity to price fluctuations. For instance, the inspection rate for spare part 8 rises consistently from 76.96% to 97.83% as price increases, whereas the dismantling rate for semi-finished product 2 surges dramatically from 4.07% to 96.11%, underscoring its high price elasticity. Conversely, finished product inspection remains consistently implemented across all scenarios, whereas the dismantling of nonconforming finished products declines sharply from 61.13% to 0%, indicating a strategic shift toward maximizing output value in high-price regimes. These patterns collectively highlight how pricing dynamics reconfigure operational priorities across the production system.

#### 4.3.6. Exchange Loss Analysis

Finally, the exchange loss, which directly affects the company’s reputation and the credit value of its products, is also critically important. Particularly in the event of negative publicity, it is necessary to increase the exchange loss to maintain credibility. Conversely, when facing economic constraints, it may be necessary to reduce the exchange loss to help salvage the company. Therefore, the results of uncertainty adjustments to the exchange loss need to be discussed, and recommendations should be provided. Similarly, after running the model 100 times, the results are presented in Table 16.

Furthermore, analysis of Table 16 indicates that fluctuations in swap losses within a ±20% range of the baseline induce considerable variability in inspection and dismantling decisions across the production process. While finished product inspection remains consistently implemented across all scenarios, multiple inspection and dismantling activities exhibit non-monotonic and at times pronounced shifts in response to changing swap loss levels. For instance, the inspection rate for spare part 6 rises sharply from 5.13% at the baseline to 88.68% at a +20% swap loss, reflecting heightened sensitivity to loss escalation. Similarly, the dismantling rate of unqualified finished products climbs markedly from 62.65% to 97.46% as swap losses increase from 0% to 20%, underscoring the growing economic incentive to mitigate quality-related losses under such conditions. These patterns collectively illustrate how swap loss magnitudes reconfigure operational priorities in a non-linear fashion across the system.

In summary, the proposed production system demonstrates remarkable dynamic adjustment capabilities when confronted with fluctuations in key parameters. Our comprehensive sensitivity analysis, which directly addresses the practical concerns regarding inspection accuracy and market uncertainty, confirms the system’s robustness. Through the implementation of the Improved Grey Wolf Optimization (IGWO) algorithm, the system achieves a precise equilibrium between inspection costs and quality output. The IGWO mechanism enables intelligent adaptation of quality control strategies: under favorable circumstances with rising prices (a proxy for demand stability), the system autonomously reduces intermediate inspection investments while prioritizing final output; conversely, when facing elevated swap losses (reflecting higher quality failure costs), it intensifies front-end inspection. Empirical evidence demonstrates that this IGWO-optimized framework maintains superior performance across diverse parameter scenarios, effectively validating the model’s practical utility despite its foundational assumptions. This intelligent, algorithm-based adaptive optimization framework provides robust technical support for precision management in modern manufacturing systems.

## 5. Conclusions

This research addresses quality–resource asymmetries in complex production systems through a systemic framework integrating the MsPIO model with a two-stage sampling mechanism. Experimental results demonstrate that confidence levels serve as a critical calibration parameter for defect-rate estimation symmetry, with the 95% confidence strategy achieving an optimal profit of 43,800 by harmonizing inspection expenditure with quality assurance. Sensitivity analyses further reveal that finished product price increases can yield nearly tenfold profit improvements, while the system maintains robust profitability under fluctuating swap loss conditions by adaptively modulating inspection intensity. The proposed IGWO algorithm effectively synchronizes multi-process decisions, underscoring the model’s systemic adaptability and offering a quantifiable bio-inspired intelligence mechanism suitable for smart manufacturing environments.

Guided by parameter sensitivity findings under uncertainty—which validate the model’s robustness to variations in defect-rate confidence and economic parameters, thereby addressing concerns related to inspection accuracy and stochastic sales—we propose a layered optimization strategy for industrial implementation. It should be acknowledged that the current framework operates under idealized assumptions, such as perfect inspection and deterministic sales; however, the sensitivity analyses confirm that the core decision logic remains effective under a wide range of realistic deviations. Enterprises may thus confidently calibrate confidence levels to sustain process stability through symmetric defect-rate estimation. In markets with high premium potential, inspection standards for intermediate products may be strategically relaxed, reallocating resources toward finished product quality and delivery efficiency. Conversely, during high-risk phases characterized by increased replacement or conversion losses, front-end inspection mechanisms should be systematically reinforced. Importantly, the model’s flexible inspection–dismantling framework enables dynamic cost–benefit adjustment, offering significant value for intelligent discrete manufacturing systems. Future research will incorporate real-time pricing mechanisms and explicitly model inspection errors and demand stochasticity to further strengthen market–production synchronization within an integrated systemic framework.

## Figures and Tables

**Table 1:** Symbol Description.

Variable	Definition
$D_{k}$	0-1 variable, used to determine whether to test spare parts or whether to test and disassemble finished products
$C_{lg}$	Purchase cost of spare parts
$Q_{i}$	Quantity of the *i*-th spare part
$u_{i}$	Purchase unit price of the *i*-th spare part
$C_{lj}$	Inspection cost of spare parts
$d_{i}$	Inspection cost of the *i*-th spare part
$C_{cz}$	Assembly cost of finished products
$C_{cs}$	Sales revenue of finished products
$Q_{c}$	Quantity of finished products
$a_{c}$	Assembly cost per finished product
$S_{c}$	Market price per finished product
$C_{cj}$	Inspection cost of finished products
$d_{c}$	Inspection cost per finished product
$P_{c}$	Defect rate of finished products
$C_{cc}$	Disassembly cost of defective finished products
$l_{c}$	Disassembly cost per defective finished product
$C_{cd}$	Exchange cost of finished products
$C_{ct}$	Return cost of finished products
$e_{c}$	Exchange cost per finished product
$r_{c}$	Return cost per finished product
$C_{bz}$	Assembly cost of semi-finished products
$Q_{b}$	Quantity of the *b*-th semi-finished product
$a_{b}$	Assembly cost of the *b*-th semi-finished product
$C_{bj}$	Inspection cost of semi-finished products
$d_{b}$	Inspection cost of the *b*-th semi-finished product
$C_{bc}$	Disassembly cost of defective semi-finished products
$P_{b}$	Defect rate of the *b*-th defective semi-finished product
$l_{b}$	Disassembly cost of the *b*-th defective semi-finished product
Z	Total cost

**Table 2:** Results for Various Production Scenarios.

	Defect Rate Interval of Semi-Finished Product 1	Defect Rate Interval of Semi-Finished Product 2	Defect Rate Interval of Semi-Finished Product 3	Defect Rate Interval of Finished Product
Production scenario1	×	×	×	[0.1, 0.356]
Production scenario 2	×	×	×	[0.2, 0.390]
Production scenario 3	×	×	×	[0.1, 0.356]
Production scenario 4	×	×	×	[0.2, 0.390]
Production scenario 5	×	×	×	[0.1, 0.356]
Production scenario 6	×	×	×	[0.05, 0.320]
Multi-process production inspection scenario	[0, 0.455]	[0, 0.455]	[0, 0.356]	[0, 0.828]

**Table 3:** CEC2022 Test Table.

	No.	Functions	$F_{i}^{*}$
Unimodal function	1	Shifted and Fully Rotated Zakharov Function	300
Basic functions	2	Shifted and Fully Rotated Rosenbrock’s Function	400
	3	Shifted and Fully Rotated Expanded Schaffer’s $f_{6}$ Function	600
	4	Shifted and Fully Rotated Non-Continuous Rastrigin’s Function	800
	5	Shifted and Fully Rotated Levy Function	900
Hybrid functions	6	Hybrid Function 1 (*N* = 3)	1800
	7	Hybrid Function 2 (*N* = 6)	2000
	8	Hybrid Function 3 (*N* = 5)	2200
Composition functions	9	Composition Function 1 (*N* = 5)	2300
	10	Composition Function 2 (*N* = 4)	2400
	11	Composition Function 3 (*N* = 5)	2600
	12	Composition Function 4 (*N* = 6)	2700
Search range: ${\left[-100,100\right]}^{D}$			

**Table 4:** Initial Parameter Settings of Algorithms.

Algorithms	Parameter
IGWO	$\eta_{c}=2$
GWO	No fixed initial parameters
hGWOA	No fixed initial parameters
RSMGWO	$\alpha_{1}=2,B_{a}=1$
DE	F=0.5,CR=0.5
CMA-ES	No fixed initial parameters

**Table 5:** The Results of Each Element on the CEC2022 Test (Dim = 10).

Function	IGWO			GWO			GWO-LHS			GWO-SBX			GWO-OL		
	Best	Avg	Std	Best	Avg	Std	Best	Avg	Std	Best	Avg	Std	Best	Avg	Std
F1	3.01 × 10^2^	3.40 × 10^2^	35.1	4.12 × 10^2^	1.68 × 10^3^	1.64 × 10^3^	3.77 × 10^2^	1.69 × 10^3^	1.74 × 10^3^	3.00 × 10^2^	3.54 × 10^2^	31.7	3.72 × 10^2^	4.17 × 10^2^	33.3
F2	4.00 × 10^2^	4.10 × 10^2^	14.2	4.03 × 10^2^	4.20 × 10^2^	19.2	4.00 × 10^2^	4.18 × 10^2^	23.7	4.07 × 10^2^	4.29 × 10^2^	32.7	4.05 × 10^2^	4.24 × 10^2^	24.6
F3	6.00 × 10^2^	6.00 × 10^2^	0.142	6.00 × 10^2^	6.00 × 10^2^	0.305	6.00 × 10^2^	6.01 × 10^2^	1.51	6.00 × 10^2^	6.00 × 10^2^	0.475	6.00 × 10^2^	6.00 × 10^2^	0.351
F4	8.02 × 10^2^	8.09 × 10^2^	4.36	8.06 × 10^2^	8.13 × 10^2^	6.42	8.06 × 10^2^	8.15 × 10^2^	8.16	8.06 × 10^2^	8.16 × 10^2^	9.81	8.04 × 10^2^	8.14 × 10^2^	9.76
F5	9.00 × 10^2^	9.08 × 10^2^	14.0	9.00 × 10^2^	9.06 × 10^2^	8.36	9.00 × 10^2^	9.01 × 10^2^	0.648	9.00 × 10^2^	9.01 × 10^2^	0.720	9.00 × 10^2^	9.03 × 10^2^	6.01
F6	2.22 × 10^3^	5.35 × 10^3^	2.33 × 10^3^	2.24 × 10^3^	5.39 × 10^3^	2.62 × 10^3^	2.57 × 10^3^	6.62 × 10^3^	2.08 × 10^3^	2.14 × 10^3^	5.61 × 10^3^	2.78 × 10^3^	2.10 × 10^3^	4.48 × 10^3^	2.75 × 10^3^
F7	2.02 × 10^3^	2.03 × 10^3^	7.96	2.02 × 10^3^	2.04 × 10^3^	11.8	2.02 × 10^3^	2.04 × 10^3^	14.2	2.00 × 10^3^	2.02 × 10^3^	10.8	2.01 × 10^3^	2.03 × 10^3^	10.4
F8	2.20 × 10^3^	2.22 × 10^3^	8.77	2.22 × 10^3^	2.23 × 10^3^	2.07	2.20 × 10^3^	2.22 × 10^3^	10.1	2.22 × 10^3^	2.23 × 10^3^	1.87	2.22 × 10^3^	2.22 × 10^3^	2.94
F9	2.53 × 10^3^	2.53 × 10^3^	12.8	2.53 × 10^3^	2.56 × 10^3^	26.3	2.53 × 10^3^	2.55 × 10^3^	27.3	2.53 × 10^3^	2.54 × 10^3^	46.4	2.53 × 10^3^	2.53 × 10^3^	12.7
F10	2.50 × 10^3^	2.53 × 10^3^	54.8	2.50 × 10^3^	2.57 × 10^3^	58.6	2.50 × 10^3^	2.59 × 10^3^	49.9	2.50 × 10^3^	2.56 × 10^3^	58.4	2.50 × 10^3^	2.51 × 10^3^	15.0
F11	2.60 × 10^3^	2.70 × 10^3^	1.46 × 10^2^	2.73 × 10^3^	2.92 × 10^3^	1.55 × 10^2^	2.60 × 10^3^	2.95 × 10^3^	1.69 × 10^2^	2.60 × 10^3^	2.86 × 10^3^	1.09 × 10^2^	2.60 × 10^3^	2.75 × 10^3^	1.57 × 10^2^
F12	2.86 × 10^3^	2.86 × 10^3^	0.916	2.86 × 10^3^	2.87 × 10^3^	5.06	2.86 × 10^3^	2.87 × 10^3^	6.56	2.86 × 10^3^	2.86 × 10^3^	1.82	2.86 × 10^3^	2.86 × 10^3^	0.616

**Table 6:** Wilcoxon Test of Each Element.

	IGWO	GWO	GWO-LHS	GWO-SBX	GWO-OL
IGWO		2.44 × 10^−3^	4.88 × 10^−3^	2.69 × 10^−2^	0.470
GWO	2.44 × 10^−3^		0.470	0.176	1.22 × 10^−2^
GWO-LHS	4.88 × 10^−3^	0.470		6.40 × 10^−2^	6.40 × 10^−2^
GWO-SBX	2.69 × 10^−2^	0.176	6.40 × 10^−2^		0.301
GWO-OL	0.470	1.22 × 10^−2^	6.40 × 10^−2^	0.301	

**Table 7:** The Results of Different Algorithms on the CEC2022 Test (Dim = 10).

Function	IGWO			GWO			hGWOA			RSMGWO		
	Best	Avg	Std	Best	Avg	Std	Best	Avg	Std	Best	Avg	Std
F1	**3.13 × 10^2^**	**3.48 × 10^2^**	36.0	4.03 × 10^2^	1.22 × 10^3^	1.24 × 10^3^	3.02 × 10^2^	8.37 × 10^2^	8.72 × 10^2^	1.84 × 10^3^	4.91 × 10^3^	2.12 × 10^3^
F2	**4.06 × 10^2^**	**4.09 × 10^2^**	1.82	4.02 × 10^2^	4.24 × 10^2^	25.2	4.00 × 10^2^	4.27 × 10^2^	36.6	4.17 × 10^2^	4.26 × 10^2^	9.90
F3	**6.00 × 10^2^**	**6.00 × 10^2^**	0.208	**6.00 × 10^2^**	6.01 × 10^2^	1.20	**6.00 × 10^2^**	6.02 × 10^2^	3.44	6.10 × 10^2^	6.16 × 10^2^	5.20
F4	**8.04 × 10^2^**	8.13 × 10^2^	8.01	8.06 × 10^2^	**8.10 × 10^2^**	3.12	8.14 × 10^2^	8.33 × 10^2^	12.1	8.45 × 10^2^	8.61 × 10^2^	13.7
F5	**9.00 × 10^2^**	**9.01 × 10^2^**	0.727	**9.00 × 10^2^**	9.05 × 10^2^	13.1	9.01 × 10^2^	1.01 × 10^3^	1.20 × 10^2^	9.60 × 10^2^	1.08 × 10^3^	1.81 × 10^2^
F6	**2.00 × 10^3^**	4.73 × 10^3^	2.96 × 10^3^	3.39 × 10^3^	6.43 × 10^3^	2.12 × 10^3^	2.21 × 10^3^	**4.60 × 10^3^**	2.16 × 10^3^	2.79 × 10^4^	4.85 × 10^5^	4.05 × 10^5^
F7	**2.00 × 10^3^**	**2.02 × 10^3^**	7.42	2.02 × 10^3^	2.03 × 10^3^	7.94	2.02 × 10^3^	2.03 × 10^3^	8.80	2.04 × 10^3^	2.05 × 10^3^	10.8
F8	**2.20 × 10^3^**	**2.22 × 10^3^**	10.6	2.22 × 10^3^	2.23 × 10^3^	2.09	2.22 × 10^3^	**2.22 × 10^3^**	1.85	2.23 × 10^3^	2.23 × 10^3^	2.14
F9	**2.53 × 10^3^**	**2.53 × 10^3^**	0.389	**2.53 × 10^3^**	2.55 × 10^3^	24.6	**2.53 × 10^3^**	2.54 × 10^3^	20.2	**2.53 × 10^3^**	2.55 × 10^3^	27.8
F10	**2.50 × 10^3^**	**2.52 × 10^3^**	46.4	**2.50 × 10^3^**	2.57 × 10^3^	58.3	**2.50 × 10^3^**	2.56 × 10^3^	63.4	**2.50 × 10^3^**	**2.52 × 10^3^**	54.6
F11	**2.60 × 10^3^**	**2.69 × 10^3^**	1.25 × 10^2^	2.91 × 10^3^	2.97 × 10^3^	92.2	**2.60 × 10^3^**	2.74 × 10^3^	1.48 × 10^2^	2.76 × 10^3^	2.80 × 10^3^	18.0
F12	**2.86 × 10^3^**	**2.86 × 10^3^**	1.42	**2.86 × 10^3^**	**2.86 × 10^3^**	0.981	**2.86 × 10^3^**	2.88 × 10^3^	21.7	**2.86 × 10^3^**	**2.86 × 10^3^**	0.888

**Table 8:** Wilcoxon Test of Different Algorithms.

	IGWO	GWO	hGWOA	RSMGWO
IGWO		2.44 × 10^−3^	2.69 × 10^−2^	1.47 × 10^−3^
GWO	2.44 × 10^−3^		0.677	0.110
hGWOA	2.69 × 10^−2^	0.677		4.25 × 10^−2^
RSMGWO	1.47 × 10^−3^	0.110	4.25 × 10^−2^	

**Table 9:** The Results of Different Global Optimization Methods on the CEC2022 Test (Dim = 10).

Function	IGWO			DE			CMA-ES		
	Best	Avg	Std	Best	Avg	Std	Best	Avg	Std
F1	3.01 × 10^2^	3.40 × 10^2^	35.1	7.63 × 10^2^	1.59 × 10^3^	4.76 × 10^2^	3.00 × 10^2^	9.50 × 10^2^	8.42 × 10^2^
F2	4.00 × 10^2^	4.10 × 10^2^	14.2	4.00 × 10^2^	4.04 × 10^2^	3.14	4.00 × 10^2^	4.11 × 10^2^	7.21
F3	6.00 × 10^2^	6.00 × 10^2^	0.142	6.00 × 10^2^	6.00 × 10^2^	7.12× 10^−2^	6.00 × 10^2^	6.00 × 10^2^	1.27 × 10^−4^
F4	8.02 × 10^2^	8.09 × 10^2^	4.36	8.11 × 10^2^	8.13 × 10^2^	2.86	8.01 × 10^2^	8.03 × 10^2^	1.75
F5	9.00 × 10^2^	9.08 × 10^2^	14.0	9.00 × 10^2^	9.00 × 10^2^	0.344	9.00 × 10^2^	9.00 × 10^2^	0.00
F6	2.22 × 10^3^	5.35 × 10^3^	2.33 × 10^3^	1.81 × 10^3^	2.17 × 10^3^	5.04 × 10^2^	1.87 × 10^3^	3.72 × 10^3^	1.91 × 10^3^
F7	2.02 × 10^3^	2.03 × 10^3^	7.96	2.00 × 10^3^	2.00 × 10^3^	2.50	2.02 × 10^3^	2.04 × 10^3^	45.1
F8	2.20 × 10^3^	2.22 × 10^3^	8.77	2.21 × 10^3^	2.22 × 10^3^	5.74	2.22 × 10^3^	2.24 × 10^3^	37.1
F9	2.53 × 10^3^	2.53 × 10^3^	12.8	2.53 × 10^3^	2.53 × 10^3^	1.68	2.54 × 10^3^	2.57 × 10^3^	38.3
F10	2.50 × 10^3^	2.53 × 10^3^	54.8	2.40 × 10^3^	2.41 × 10^3^	24.6	2.50 × 10^3^	2.55 × 10^3^	55.4
F11	2.60 × 10^3^	2.70 × 10^3^	1.46 × 10^2^	2.90 × 10^3^	3.11 × 10^3^	1.79 × 10^2^	2.60 × 10^3^	2.87 × 10^3^	94.9
F12	2.86 × 10^3^	2.86 × 10^3^	0.916	2.86 × 10^3^	2.87 × 10^3^	0.936	2.86 × 10^3^	2.87 × 10^3^	0.942

**Table 10:** Wilcoxon Test of Different Global Optimization Methods.

	IGWO	DE	CMA-ES
IGWO		0.424	0.151
DE	0.424		0.470
CMA-ES	0.151	0.470	

**Table 11:** Results of Various Situations in a Single Process.

	Inspecting Spare Parts 1	Inspecting Spare Parts 2	Inspecting Finished Products	Disassembling Unqualified Finished Products	Total Profit (The Opposite of Total Cost)
Case 1	0.16%	14.84%	100.00%	100.00%	10,916
Case 2	19.71%	1.10%	100.00%	100.00%	8324
Case 3	30.52%	0.07%	100.00%	100.00%	10,700
Case 4	4.95%	37.22%	100.00%	100.00%	9045
Case 5	3.70%	6.14%	100.00%	100.00%	11,145
Case 6	22.90%	79.66%	12.16%	18.52%	12,026

**Table 12:** Results of Situation in a Multi-process.

	IGWO	GWO	hGWOA	RSMGWO
Inspecting spare parts 1	39.94%	58.13%	47.00%	55.80%
Inspecting spare parts 2	21.16%	30.53%	70.03%	3.06%
Inspecting spare parts 3	27.00%	14.67%	60.98%	39.60%
Inspecting spare parts 4	48.57%	6.13%	67.83%	2.44%
Inspecting spare parts 5	22.59%	11.01%	26.16%	63.23%
Inspecting spare parts 6	5.13%	80.00%	91.08%	22.03%
Inspecting spare parts 7	22.12%	17.70%	74.66%	45.08%
Inspecting spare parts 8	34.64%	9.27%	10.47%	81.59%
Inspecting semi-finished products 1	40.72%	6.73%	89.79%	33.46%
Inspecting semi-finished products 2	28.53%	54.90%	38.25%	15.25%
Inspecting semi-finished products 3	27.37%	12.63%	53.49%	49.28%
Dismantling semi-finished products 1	16.09%	50.90%	47.25%	0.45%
Dismantling semi-finished products 2	1.19%	11.85%	61.62%	87.11%
Dismantling semi-finished products 3	65.60%	79.23%	79.91%	9.90%
Inspecting finished products	100.00%	100.00%	100.00%	100.00%
Dismantling of unqualified finished products	62.65%	70.13%	88.61%	56.66%
Total profit (The opposite of total cost)	43,800	42,800	42,800	43,400

**Table 13:** Estimated defective rate at different confidence levels.

Confidence Level	15%	35%	55%	75%
Part 1	[0.000, 0.038]	[0.000, 0.050]	[0.000, 0.066]	[0.000, 0.090]
Part 2	[0.000, 0.038]	[0.000, 0.050]	[0.000, 0.066]	[0.000, 0.090]
Part 3	[0.000, 0.038]	[0.000, 0.050]	[0.000, 0.066]	[0.000, 0.090]
Part 4	[0.000, 0.038]	[0.000, 0.050]	[0.000, 0.066]	[0.000, 0.090]
Part 5	[0.000, 0.038]	[0.000, 0.050]	[0.000, 0.066]	[0.000, 0.090]
Part 6	[0.000, 0.038]	[0.000, 0.050]	[0.000, 0.066]	[0.000, 0.090]
Part 7	[0.000, 0.038]	[0.000, 0.050]	[0.000, 0.066]	[0.000, 0.090]
Part 8	[0.000, 0.038]	[0.000, 0.050]	[0.000, 0.066]	[0.000, 0.090]
Semi-finished product 1	[0.100, 0.199]	[0.100, 0.228]	[0.100, 0.267]	[0.100, 0.322]
Semi-finished product 2	[0.100, 0.199]	[0.100, 0.228]	[0.100, 0.267]	[0.100, 0.322]
Semi-finished product 3	[0.100, 0.167]	[0.100, 0.188]	[0.100, 0.215]	[0.100, 0.255]
Finished Product	[0.100, 0.519]	[0.100, 0.564]	[0.100, 0.620]	[0.100, 0.692]

**Table 14:** Average Decision Results at Different Confidence Levels.

Confidence Level	15%	35%	55%	75%
Inspecting spare parts 1	59.43%	13.61%	25.20%	22.16%
Inspecting spare parts 2	62.92%	52.89%	15.08%	15.04%
Inspecting spare parts 3	55.71%	7.48%	59.86%	39.56%
Inspecting spare parts 4	34.29%	25.45%	67.40%	2.89%
Inspecting spare parts 5	55.82%	25.27%	65.29%	16.39%
Inspecting spare parts 6	88.68%	30.75%	81.28%	15.60%
Inspecting spare parts 7	74.23%	33.93%	34.52%	31.65%
Inspecting spare parts 8	96.22%	39.92%	30.52%	15.25%
Inspecting semi-finished products 1	8.58%	13.90%	20.93%	83.82%
Inspecting semi-finished products 2	14.73%	42.25%	28.72%	5.22%
Inspecting semi-finished products 3	4.56%	76.79%	20.49%	4.52%
Dismantling semi-finished products 1	30.07%	10.35%	60.80%	37.94%
Dismantling semi-finished products 2	46.45%	43.32%	57.60%	62.97%
Dismantling semi-finished products 3	21.13%	1.48%	60.16%	48.44%
Inspecting finished products	100.00%	100.00%	100.00%	100.00%
Dismantling of unqualified finished products	97.46%	71.98%	48.19%	35.39%
Total profit (The opposite of total cost)	43,400	41,600	41,200	43,600

**Table 15:** Average Decision Results for Different Finished Product Selling Prices.

Finished Product Price	−40%	−20%	0%	20%	40%
Inspecting spare parts 1	44.27%	33.77%	39.94%	36.15%	59.86%
Inspecting spare parts 2	30.81%	19.91%	21.16%	11.93%	36.28%
Inspecting spare parts 3	46.46%	73.99%	27.00%	42.43%	66.98%
Inspecting spare parts 4	76.88%	13.91%	48.57%	45.34%	34.99%
Inspecting spare parts 5	56.89%	41.53%	22.59%	54.54%	43.67%
Inspecting spare parts 6	19.30%	45.74%	5.13%	24.29%	60.02%
Inspecting spare parts 7	22.13%	61.12%	22.12%	35.46%	2.36%
Inspecting spare parts 8	76.96%	73.98%	34.64%	63.71%	97.83%
Inspecting semi-finished products 1	36.67%	82.37%	40.72%	0.00%	4.35%
Inspecting semi-finished products 2	55.19%	34.79%	28.53%	20.05%	0.00%
Inspecting semi-finished products 3	22.41%	16.57%	27.37%	53.53%	13.64%
Dismantling semi-finished products 1	22.02%	12.13%	16.09%	35.62%	34.83%
Dismantling semi-finished products 2	4.07%	31.73%	1.19%	84.45%	96.11%
Dismantling semi-finished products 3	64.97%	50.49%	65.60%	94.79%	52.10%
Inspecting finished products	100.00%	100.00%	100.00%	100.00%	100.00%
Dismantling of unqualified finished products	61.13%	59.66%	62.65%	7.30%	0.00%
Total profit (The opposite of total cost)	7920	24,600	43,800	59,640	78,480

**Table 16:** Average Decision Results with Different Swap Losses.

Swap Losses	−20%	−10%	0%	10%	20%
Inspecting spare parts 1	78.75%	47.15%	39.94%	40.38%	59.43%
Inspecting spare parts 2	3.31%	55.16%	21.16%	63.62%	62.92%
Inspecting spare parts 3	38.68%	74.12%	27.00%	18.60%	55.71%
Inspecting spare parts 4	88.42%	67.84%	48.57%	6.96%	34.29%
Inspecting spare parts 5	12.65%	66.59%	22.59%	70.70%	55.82%
Inspecting spare parts 6	14.57%	69.37%	5.13%	19.51%	88.68%
Inspecting spare parts 7	12.89%	56.46%	22.12%	22.46%	74.23%
Inspecting spare parts 8	84.60%	36.44%	34.64%	55.62%	96.22%
Inspecting semi-finished products 1	7.49%	23.91%	40.72%	0.31%	8.58%
Inspecting semi-finished products 2	5.26%	20.02%	28.53%	11.85%	14.73%
Inspecting semi-finished products 3	3.26%	36.58%	27.37%	7.54%	4.56%
Dismantling semi-finished products 1	81.99%	32.15%	16.09%	62.91%	30.07%
Dismantling semi-finished products 2	53.18%	32.48%	1.19%	17.30%	46.45%
Dismantling semi-finished products 3	50.22%	8.19%	65.60%	29.49%	21.13%
Inspecting finished products	100.00%	100.00%	100.00%	100.00%	100.00%
Dismantling of unqualified finished products	14.04%	19.61%	62.65%	41.70%	97.46%

## Data Availability

The data used in this study are available from the corresponding author upon reasonable request.

## Appendix A

**Table A1:** Single-process Production Scenario.

Scenario	Spare Part 1			Spare Part 2			Finished Product				Defective Finished Product	
	Defect Rate	Purchase Unit Price	Inspection Cost	Defect Rate	Purchase Unit Price	Inspection Cost	Defect Rate	Assembly Cost	Inspection Cost	Market Price	Exchange Cost	Disassembly Cost
1	10%	4	2	10%	18	3	10%	6	3	56	6	5
2	20%	4	2	20%	18	3	20%	6	3	56	6	5
3	10%	4	2	10%	18	3	10%	6	3	56	30	5
4	20%	4	1	20%	18	1	20%	6	2	56	30	5
5	10%	4	8	20%	18	1	10%	6	2	56	10	5
6	5%	4	2	5%	18	3	5%	6	3	56	10	40

**Table A2:** Multi-process Production Scenario.

Spare Part	Defect Rate	Purchase Unit Price	Inspection Cost	Semi-Finished Product	Defect Rate	Assembly Cost	Inspection Cost	Disassembly Cost
1	10%	2	1	1	10%	8	4	6
2	10%	8	1	2	10%	8	4	6
3	10%	12	2	3	10%	8	4	6
4	10%	2	1					
5	10%	8	1	Finished product	10%	8	6	10
6	10%	12	2					
7	10%	8	1		Market price		Exchange cost	
8	10%	12	2	Finished product	200		40	
